# *Pseudomonas aeruginosa* in chronic lung disease: untangling the dysregulated host immune response

**DOI:** 10.3389/fimmu.2024.1405376

**Published:** 2024-06-28

**Authors:** Rhea Nickerson, Christina S. Thornton, Brent Johnston, Amy H. Y. Lee, Zhenyu Cheng

**Affiliations:** ^1^ Department of Microbiology and Immunology, Faculty of Medicine, Dalhousie University, Halifax, NS, Canada; ^2^ Department of Medicine, Cumming School of Medicine, University of Calgary, Calgary, AB, Canada; ^3^ Department of Microbiology, Immunology and Infectious Diseases, Cumming School of Medicine, University of Calgary, Calgary, AB, Canada; ^4^ Department of Molecular Biology and Biochemistry, Faculty of Science, Simon Fraser University, Burnaby, BC, Canada

**Keywords:** *Pseudomonas aeruginosa*, chronic lung disease, inflammation, neutrophils, innate immune response, adaptive immune response

## Abstract

*Pseudomonas aeruginosa* is a highly adaptable opportunistic pathogen capable of exploiting barriers and immune defects to cause chronic lung infections in conditions such as cystic fibrosis. In these contexts, host immune responses are ineffective at clearing persistent bacterial infection, instead driving a cycle of inflammatory lung damage. This review outlines key components of the host immune response to chronic *P. aeruginosa* infection within the lung, beginning with initial pathogen recognition, followed by a robust yet maladaptive innate immune response, and an ineffective adaptive immune response that propagates lung damage while permitting bacterial persistence. Untangling the interplay between host immunity and chronic *P. aeruginosa* infection will allow for the development and refinement of strategies to modulate immune-associated lung damage and potentiate the immune system to combat chronic infection more effectively.

## 1 Introduction

*Pseudomonas aeruginosa* is a highly adaptable Gram-negative bacterium which is prevalent in the environment and causes a wide range of opportunistic infections in humans. *P. aeruginosa* can infect diverse tissues, including the lung, where it can cause acute pneumonia or chronic infections that exacerbate the pathology of conditions including cystic fibrosis (CF), non-CF bronchiectasis (NCFB) and chronic obstructive pulmonary disease (COPD) (1–4)*. P. aeruginosa* is one of the most common and fatal multi-drug resistant bacteria mediating acute pneumonia in hospital settings (5–7). In the context of chronic infections, *P. aeruginosa* is the most prevalent pathogen in adult persons with CF (pwCF), with recent registry data in the USA, UK, and Canada reporting up to 40-50% are chronically colonized, despite significant therapeutic advances over the last decade (8–11). Chronic *P. aeruginosa* infection is associated with adverse outcomes and greater mortality in pwCF. *P. aeruginosa*’s role in other chronic lung conditions remains understudied, though reports are beginning to highlight its importance in the pathology and outcomes of these diseases as well. *P. aeruginosa* is able to chronically colonize the lungs of COPD patients, in a similar manner to pwCF, where it has been associated with increased respiratory symptoms and inflammatory responses in the airway (2, 4). Similar findings have been reported for NCFB, where *P. aeruginosa* colonization has been recorded in up to 50% of adults (4, 12). In these cohorts, *P. aeruginosa* was more likely to cause persistent infections than other common lung pathogens, and was also associated with a greater rate of hospitalizations (12). Taken together, much of the pathogenicity of chronic *P. aeruginosa* infection stems from maladaptive hyper-inflammatory host immune responses, which fail to clear infection and contribute to self-perpetuating lung damage. However, therapeutic interventions targeting chronic *P. aeruginosa* are still broadly limited to antibiotic-mediated eradication efforts, which frequently fail in chronic settings due to bacterial adaptations such as biofilm formation and increasing prevalence of multi-drug resistance (13). Better understanding the dysregulation of host immunity in chronic *P. aeruginosa* infection will lead to the development of novel therapies to modulate immune-associated lung damage and potentiate the immune system to combat chronic infection more effectively.

This review aims to outline key contexts for chronic *P. aeruginosa* infection, including host factors that influence its transition to chronicity; elements of the immune response which are either protective or pathological; and strategies to modulate chronic *P. aeruginosa* infection, including antibiotics, vaccines, immunomodulatory therapies, and cystic fibrosis transmembrane conductance regulator (CFTR) modulators.

## 2 Infection contexts

### 2.1 Acute infection

As an opportunistic pathogen, *P. aeruginosa* rarely causes infection in healthy, immunocompetent individuals, but frequently capitalizes on immune dysfunction or mechanical disruption for colonization. Reflecting this, *P. aeruginosa* is not a common cause of community-acquired pneumonia but is among the leading causes of hospital-acquired pneumonia, where it frequently causes more severe illness than other bacterial pathogens due to its array of virulence factors (5, 14–16). *P. aeruginosa* can exploit patients who are immunocompromised due to other health concerns, such as those undergoing immunotherapy, chemotherapy, or receiving mechanical ventilation (14). The most common outcomes of acute respiratory infection with *P. aeruginosa* are either bacterial clearance, by the host immune response or antibiotic treatment –or, in the event of a failure to control infection, dissemination of bacteria resulting in sepsis, or respiratory failure as an outcome of uncontrolled infection or excessive immune-mediated damage secondary to infection (16). However, in these settings, acute infections with *P. aeruginosa* rarely transition to a state of chronicity.

### 2.2 Chronic infection

**Figure 1:**
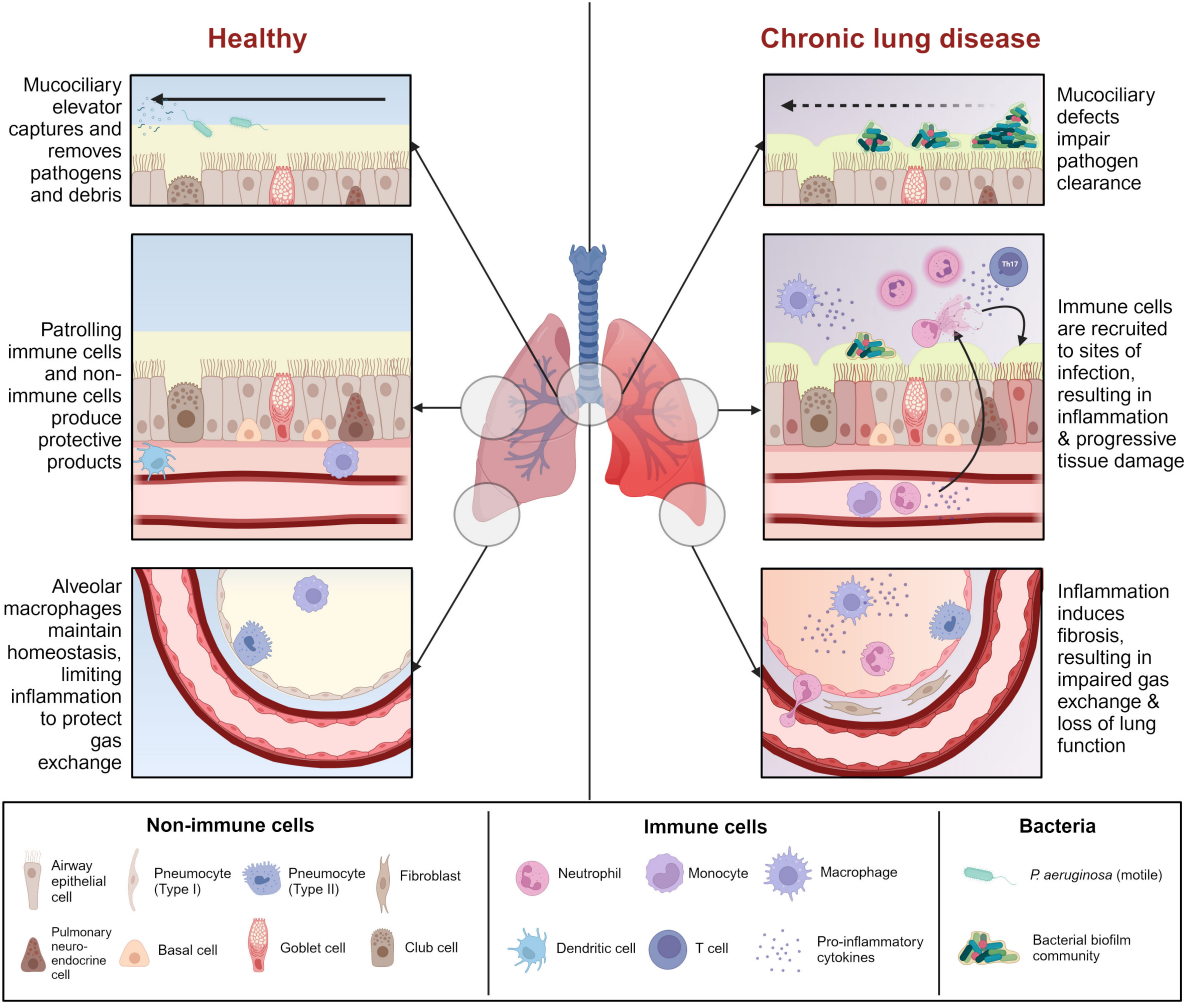
Comparison between healthy airway and chronic lung disease. Chronic lung disease is characterized by mucociliary defects (i.e., mucus dehydration, ciliary dysfunction) which impair initial clearance of pathogens from the airway. Reduced pathogen clearance promotes immune cell recruitment and activation, resulting in a cascade of inflammation and tissue damage which renders the airway more susceptible to infection. Some chronic lung diseases are characterized by pre-existing inflammation which heightens this effect. Prolonged unresolving inflammation leads to tissue remodeling and fibrosis, which affects gas exchange in the alveoli, ultimately leading to lung failure. Figure created with BioRender.com.

Chronic infections with *P. aeruginosa* occur in select circumstances, most often in patients with structural chronic lung conditions, such as CF, NCFB, and COPD. These conditions are united by defects in lung innate immunity, such as impairment of the mucociliary elevator, mucus buildup, damage to the lung epithelial barrier, and/or baseline inflammation, which render the lungs more susceptible to infection (**Figure 1**).

CF is the most common autosomal recessive genetic disease in the Caucasian population, affecting approximately 70,000 individuals worldwide (17). CF results from mutations in the cystic fibrosis transmembrane conductance receptor (CFTR), a chloride channel that coordinates ion and fluid transport across the epithelium. CFTR is found throughout the body, and although CF affects numerous organs including the pancreas, intestine, liver, reproductive tract, and sweat glands, the primary cause of morbidity and mortality in CF is respiratory failure (18). In the lungs, CFTR regulates both chloride ion absorption and secretion in distinct cell types to fine-tune the volume and hydration of airway surface liquid (ASL) (19). Mutations in *CFTR* result in reduced ASL volume and mucus dehydration in the CF airway, leading to mucus plugging and attenuated mucociliary clearance –preventing the clearance of pathogens from the lungs (20, 21). Mucus dehydration and accumulation have been shown to provide a unique niche that promotes biofilm formation by pathogens such as *P. aeruginosa*, allowing the transition towards a chronic phenotype (22, 23). *CFTR* mutations may also predispose pwCF to sterile inflammation and impair innate immune cell function, rendering them even more susceptible to infection (24–26). Establishment of chronic bacterial infection results in a self-perpetuating inflammatory response characterized by intense neutrophil recruitment and production of inflammatory mediators, which leads to irreversible airway damage and fibrosis (27). PwCF are classically colonized by specific pathogens, with *Staphylococcus aureus* predominating in children, but transitioning to *P. aeruginosa* as the dominant colonizing pathogen once individuals reach adolescence. Several adaptive features, including *P. aeruginosa*’s ability to better adjust to the progressively diseased lung environment and evade host immunity and/or directly target *S. aureus* to outcompete it, contribute to dominance of *P. aeruginosa* in the adult CF lung (28). Other classical CF pathogens include *Achromobacter xylosoxidans*, *Burkholderia cepacia* complex, *Haemophilus influenzae*, and *Stenotrophomonas maltophilia* (29, 30). However, it is becoming increasingly recognized that chronic respiratory infections in pwCF are polymicrobial, and that CF disease intrinsically perturbs the airway microbiome (31, 32). Recent metagenomic work has demonstrated that the airway microbial communities of pwCF are distinct from healthy individuals, beginning as early as 4 years old, and even in the absence of classical CF pathogens (32). Airway microbial load and composition are unique to each individual with CF, although patterns emerge based on the presence of classical CF pathogens, and the stage of lung disease (32–36). Airway commensal communities have been observed to fragment over time, correlating with the emergence and takeover of CF pathogens such as *P. aeruginosa, S. aureus*, and *S. maltophilia*. Similar trends in disruption of airway commensal diversity by pathogens like *P. aeruginosa* are beginning to be observed for other diseases such as NCFB. Only in end-stage lung disease do bacterial monocultures of dominant CF pathogens like *P. aeruginosa* take over in the lungs (32). However, it is the sheer prevalence of *P. aeruginosa* in the CF population, as well as its strong association with worsened outcomes, that has prompted significant research into chronic *P. aeruginosa* infection.

Other conditions that involve enhanced susceptibility to chronic *P. aeruginosa* infection also involve defects in the lung mucus layer, mucociliary elevator defects, and inflammatory lung damage. NCFB refers to a wide range of disease etiologies (more than 50 currently recognized) which result in inflammation, dilation, and irreversible thickening of the bronchial walls (37). These airway architectural changes are often accompanied by mucus accumulation, chronic infection of the airways by pathogens like *P. aeruginosa*, and, similar to CF, persistent neutrophilic inflammation which perpetuates a vicious cycle of airway damage (38). Only 38% of bronchiectasis cases have identifiable causes, which can include congenital causes, such as primary ciliary dyskinesis; genetic causes like alpha-1 antitrypsin deficiency; autoimmune conditions including systemic lupus erythematosus and rheumatoid arthritis; environmental or lifestyle-related causes, including long-term smoking or exposure to pollutants; or post-infectious causes, such as prior infection with various respiratory pathogens, including COVID-19 (38–40). Several idiopathic conditions such as diffuse panbronchiolitis also proceed to bronchiectasis in late-stage disease (41). Following disease onset, NCFB patients are more susceptible to respiratory infections with several key pathogens, of which *P. aeruginosa* and *H. influenzae* are the most prevalent (39). A recent study utilizing the European EMBARC registry found that in most southern European countries, over 50% of adults with NCFB were colonized with *P. aeruginosa* within a one-year period, making it the most common pathogen in this population (39). Recent culture-independent work has shown that a distinct cluster of NCFB patients are characterized by a *Pseudomonas*-dominant microbiome, characterized by lower alpha diversity and positive correlation with clinical features such as exacerbations (42, 43). As in CF, *P. aeruginosa* infection is more common in adults than children (44, 45). *P. aeruginosa* infection is considered a major cause of morbidity in all bronchiectasis patients, defined by increased exacerbations in respiratory pathology, reduced lung function, and increased mortality, particularly when infection becomes chronic (12, 46).

COPD is characterized by progressive airflow limitation, airway inflammation, emphysema, and chronic mucus production resulting from airway narrowing (47). COPD is caused by prolonged exposure to external toxic irritants, such as cigarette smoke and air pollutants, with a variety of genetic and environmental risk factors (47). There are multiple subtypes of COPD that interestingly correlate with different airway pathogen prevalences (48). *P. aeruginosa* is less common in COPD than in CF or NCFB, with an overall prevalence estimated to be approximately 4%, increasing to 13% in patients with advanced airway obstruction (2, 49, 50). However, COPD affects approximately 100 times as many individuals as CF, and is increasing in incidence, underscoring that *P. aeruginosa* infections in COPD are still a significant concern (51). *P. aeruginosa* has been shown to cause both acute infections leading to disease exacerbations, and persistent long-term infections in a subset of patients (52). COPD exacerbations caused by *P. aeruginosa* are generally more severe, and associated with higher levels of hospitalization and mortality than exacerbations caused by other identifiable pathogens (51). Generally, the COPD patients who fail to clear *P. aeruginosa* and retain long-term infections are more severely-ill than the patients with short-term infections (53). COPD patients with persistent *P. aeruginosa* infection followed over years exhibited clonal diversification of a stable infection that evolved toward characteristics such as hypermutability, increased antibiotic resistance, reduced protease production, loss of motility, and biofilm production, all signs of bacterial evolution toward chronicity (2, 54).

### 2.3 *P. aeruginosa* adaptation in chronic infection

The question of differentiating acute from chronic *P. aeruginosa* infection is one that remains challenging and sometimes leaves past experimental work in the context of “chronic” infection difficult to interpret. Chronic infection has historically been defined in clinical settings by repeated detection of *P. aeruginosa* culture-positive samples over various timeframes, often during routine testing, although there is no set standard (55). The most commonly-used guidelines are the Leeds criteria, which define chronic infection as greater than 50% *P. aeruginosa*-positive monthly cough swabs/sputum cultures within a 12-month period (56). While practical for clinical use, this definition does not actually distinguish persistent *P. aeruginosa* infection from recurrent acute infections in susceptible individuals. Given the association between chronic infection and bacterial adaptations such as antibiotic resistance, biofilm formation, and immune evasion, it is important to understand how chronic *P. aeruginosa* infections develop, and to be able to identify when infections transition into chronicity.

The development of chronic *P. aeruginosa* infection often follows a similar trajectory and is associated with various adaptations to the lung environment. Most of the work charting the evolution of chronic *P. aeruginosa* over time has been done in the CF context; however, recent findings in NCFB and COPD patients are generally converging with this work, despite a few important differences. Initial infection with *P. aeruginosa* commonly begins in childhood for pwCF (57). The disparate etiologies of NCFB mean that it can begin in childhood or adulthood, with current findings indicating that *P. aeruginosa* infections are more common in adults with NCFB. COPD affects primarily adults, and so it can be assumed that *P. aeruginosa* infections are also acquired in adulthood for COPD. *P. aeruginosa* can be acquired from environmental sources, as it is a ubiquitous environmental pathogen found in soil and water. However, *P. aeruginosa* can also be acquired from patient-to-patient transmission, which occurs more commonly in CF than NCFB or COPD (58). The *P. aeruginosa* strains able to cause patient-to-patient transmission are called clonal or epidemic strains, with the Liverpool epidemic strain (LES) being the most notable example (58). Most of these clonal strains have similar adaptations including enhanced biofilm formation and high levels of antibiotic resistance which give them colonization advantages.

Because of the strong association between *P. aeruginosa* infection and worsened lung function decline, initial *P. aeruginosa* infections are heavily treated with antibiotics and often successfully cleared. This widespread eradication therapy has led to significant decreases in *P. aeruginosa* infection rates in children and adolescents with CF, shifting the median age of chronic *P. aeruginosa* acquisition in this group (8, 57). However, these eradication efforts often eventually fail (59) as *P. aeruginosa* adapts within the lung to evade antibiotic clearance (60). Related to this, concerns are increasing regarding the rising prevalence of MDR *P. aeruginosa* strains, including clonal strains which more readily establish chronic infections due to their multi-drug resistance (61). When eradication efforts fail, this often begins an “intermittent” phase of infection, characterized by cycles of colonization, treatment, and recolonization, during which time microbiological culture samples may vary. Eventually, intermittently-colonizing *P. aeruginosa* acquires mutations and undergoes lifestyle changes which protect the bacteria from both host and antibiotic clearance, resulting in chronic infection (62).

**Figure 2:**
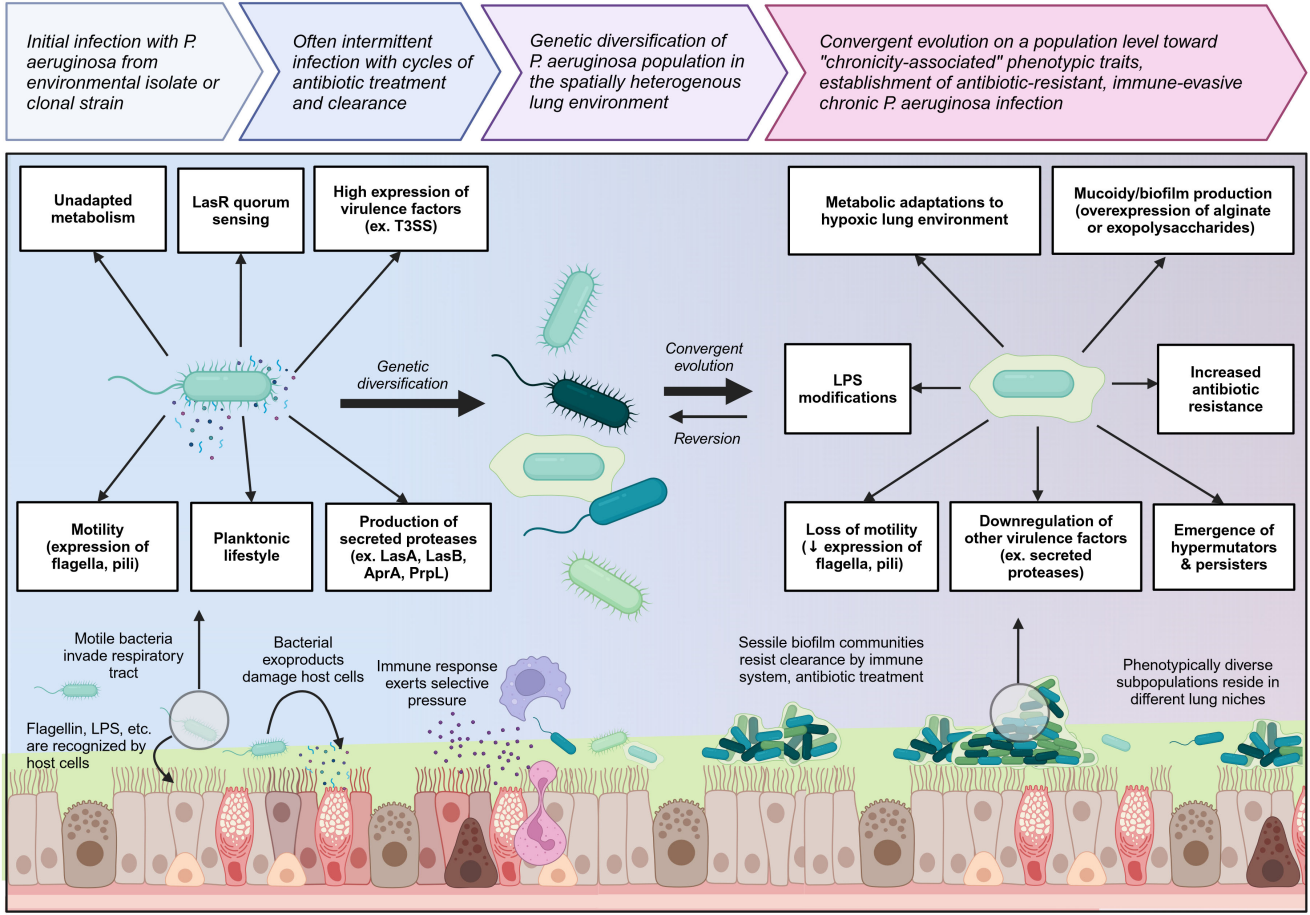
Overview of the transition from initial colonizing *P. aeruginosa* to chronic lung-adapted *P. aeruginosa*. *P. aeruginosa* generally enters the lung as a motile planktonic bacterium expressing flagella and pili and producing high levels of virulence factors and exoproducts such as secreted proteases which are immunogenic and damage the lung tissue. This prototypical acute phenotype is outlined on the left. Initial infection can occur with environmental isolates or clonal strains which may already have some degree of lung adaptation. As *P. aeruginosa* adapts to the lung environment in chronic lung diseases such as cystic fibrosis, it genetically diversifies and then converges on a population level toward a set of chronicity-associated phenotypes highlighted on the right. It loses key virulence factors and becomes less immunogenic. Transition to a sessile biofilm lifestyle as well as mucoidy increase resistance to both immune and antibiotic clearance. Biofilm communities undergo clonal diversification to give rise to hypermutators and persisters which further increase *P. aeruginosa* resilience within the lung niche. Created with BioRender.com.

Chronically-infected individuals are generally infected with a single strain of *P. aeruginosa* which persists and genetically diversifies over a prolonged period of time, leading to significant spatiotemporal diversity within different regions of the lung (58, 60, 63). Occasionally, individuals can be superinfected with new *P. aeruginosa* strains which usually outcompete or are outcompeted by the resident population (58). Longitudinal tracking of *P. aeruginosa* populations over a ten-year period has shown that during the early stages of chronic infection, the *P. aeruginosa* population rapidly diversifies and gains a wide range of mutations, but over time, these genetically diverse lineages converge upon specific chronicity-associated phenotypes which allow the bacteria to resist antibiotics, evade immune clearance, and survive in the lung environment (63). Chronicity-associated phenotypes include mucoidy resulting from overproduction of exopolysaccharides such as alginate or Psl/Pel, which favor biofilm formation, and related loss of flagellum and pili resulting in loss of motility and transition to a sessile lifestyle (55, 62, 64). Chronicity is also associated with reduced production of exoproducts such as proteases and some key virulence factors such as the type III secretion system (T3SS), but overexpression of other virulence factors such as Cif which dysregulates endocytic recycling of CFTR, reducing chloride secretion across the epithelium (65, 66). Other chronicity-related phenotypes include increased efflux pump expression; changes in lipopolysaccharide (LPS) structure which reduce antigenicity; metabolic changes allowing for survival in hypoxic mucus microenvironments and use of different nutrients available in the lung; mutation of quorum sensing regulators such as LasR; and emergence of hypermutators and persisters (**Figure 2**) (63, 67–69). However, it is important to note that although these chronicity-associated phenotypes become enriched on a population level over time within the lung, this population is composed of spatially and temporally heterogenous subpopulations which are phenotypically diverse according to the needs of their specific lung niche. Given this heterogeneity, most of these chronicity-associated phenotypes, such as mucoidy, reduced motility, altered metabolism, and reduced virulence factor expression, are reversible, allowing *P. aeruginosa* to adapt effectively to changing selective pressures within the lung.

Recent advances in sequencing technologies, bioinformatics methods, and even sample collection strategies have revealed new complexities to the ways *P. aeruginosa* evolves and diversifies during chronic infections. All of these complex factors shape the interplay between *P. aeruginosa* and host immunity in important ways, but the scope of these topics exceeds the focus of this review. Many of the topics mentioned briefly above, as well as many more we were unable to cover, have recently been thoroughly reviewed elsewhere (57, 58, 60, 69–71).

## 3 Immune response to infection

**Figure 3:**
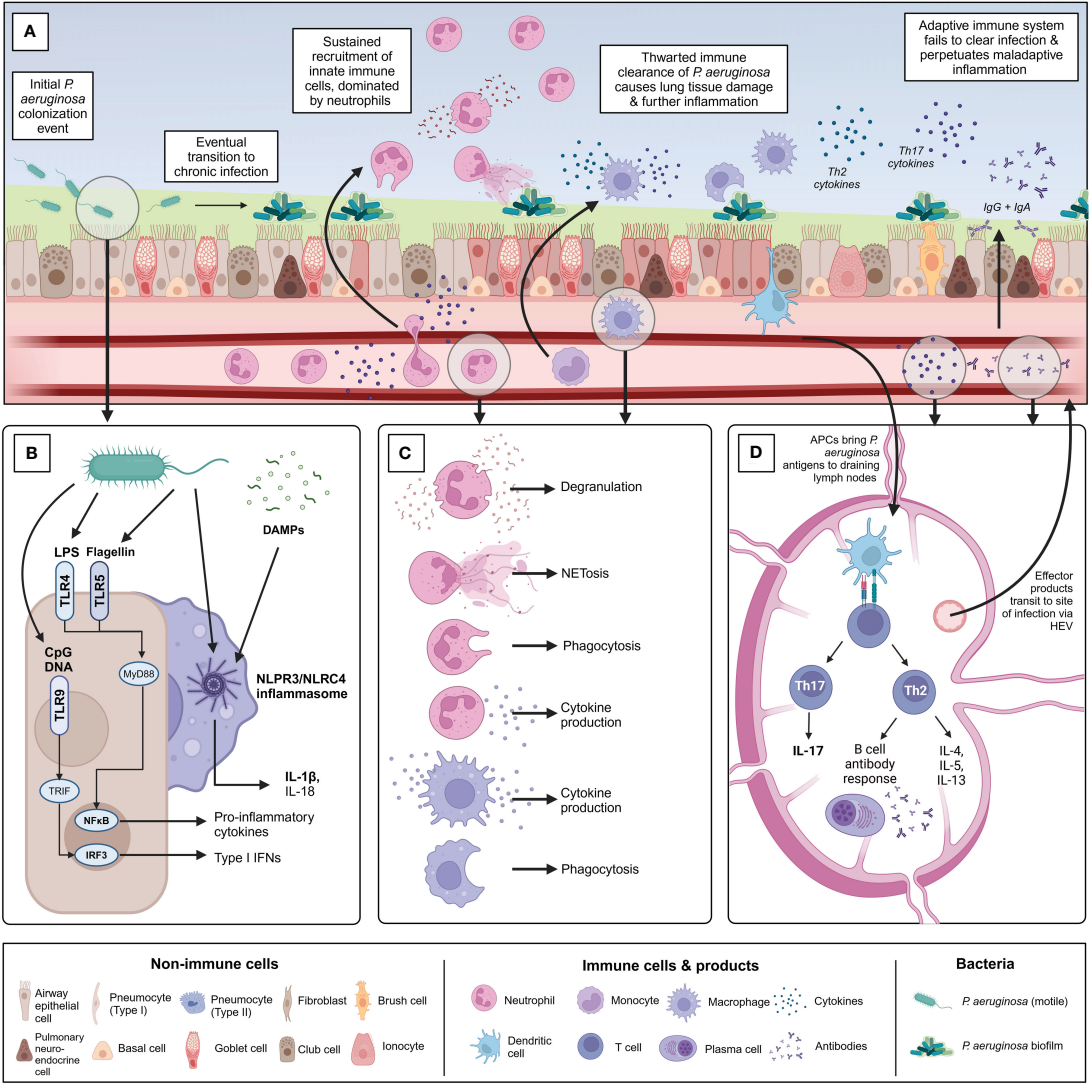
Overview of the immune response to chronic *P. aeruginosa* infection within the lung. **(A)** Summary of the timeline of chronic *P. aeruginosa* infection establishment and key immune players involved. Following initial potentially intermittent colonization, *P. aeruginosa* adapts to the diseased lung environment and establishes a biofilm community. **(B)** Overview of the primary pattern recognition receptors and pathogen-associated molecular patterns associated with initial recognition of *P. aeruginosa* by sentinel airway epithelial cells and alveolar macrophages. **(C)** Following pathogen recognition, innate immune cells are recruited to the site of infection. Chronic *P. aeruginosa* infection is characterized by neutrophilia and extensive neutrophil-mediated lung damage because of degranulation, NETosis, and cytokine production. Monocyte-derived macrophages are recruited from circulation and polarized into inflammatory “M1” macrophages at the site of infection. Interstitial macrophages and alveolar macrophages also contribute to the macrophage involvement in infection. Macrophages also contribute to production of pro-inflammatory cytokines. Phagocytosis by both macrophages and neutrophils is thwarted by chronic bacterial adaptations. **(D)** Antigen-presenting cells such as dendritic cells are activated within the lung and travel to local draining lymph nodes to activate adaptive T and B-cell mediated immunity. Naïve CD4+ T cells are predominantly polarized into Th2 and Th17 populations in chronic *P. aeruginosa* infection. Th2 cells produce characteristic cytokines IL-4, IL-5, and IL-13 and support the formation of the B cell antibody response. Robust IgG and IgA antibody production occurs in response to chronic *P. aeruginosa* infection but is largely ineffectual at clearing infection or protecting against re-infection and may contribute to further inflammation by immune complex formation. Th17 cells produce Il-17 family cytokines which indirectly support neutrophil maturation and recruitment, further contributing to detrimental neutrophil-mediated lung damage. Figure created using BioRender.com.

Chronic infection with *P. aeruginosa* is associated with significant lung damage and worsened outcomes, which is greatly compounded by a sustained and dysregulated immune response to infection, in addition to damage mediated directly by bacterial infection itself. The immune response to chronic *P. aeruginosa* is predominantly characterized by sustained neutrophilic infiltration resulting in lung damage caused by release of neutrophil effector molecules, but also involves a wide range of other cellular players such as macrophages and specific T helper cell subsets, which play various roles including contributing to this vicious cycle of neutrophilic inflammation, producing damaging mediators of their own, or preventing the formation of an effectively-balanced immune response (72). A better understanding of this dysregulated inflammatory response to infection will aid in the further development of targeted immunomodulatory therapies. Notably, it is important to recognize that much of the work investigating these dysregulated responses to chronic *P. aeruginosa* infection has been done in the context of CF, where the CFTR defect may also directly impact the functions of some immune cells, though this remains controversial (26). More work needs to be done investigating chronic *P. aeruginosa* infection in other contexts such as COPD and NCFB. Challenges in effectively modelling chronic *P. aeruginosa* infection using animal models, both within and outside the context of CF, have also added layers of complexity to unraveling context- and model-specific responses to infection (73). This section aims to introduce key pathways important for sensing and responding to *P. aeruginosa* infection, followed by the roles of innate and adaptive immune cells in mediating bacterial clearance, host damage, and regulating the immune response (**Figure 3**).

### 3.1 Pathogen recognition

Various germline-encoded pathways are utilized by non-immune cells such as airway epithelial cells, and innate immune cells such as macrophages and neutrophils, in the early sensing of *P. aeruginosa* as it enters the respiratory tract. The pathways governing initial recognition of *P. aeruginosa* are vital for directing the subsequent inflammatory response in both acute and chronic infection, and there are several ways these pathways become dysregulated when infection becomes chronic.

#### 3.1.1 TLR signaling

When *P. aeruginosa* enters the lung, the first cells it encounters will likely be airway epithelial cells, as well as various sentinel immune cells, from dendritic cells (DCs) and mast cells in the upper airways, to alveolar macrophages in the alveoli. These various cell types play critical roles in the initial recognition and shaping of the inflammatory response to pathogens, which occurs in the first minutes, hours, and days post-infection. Sustained pathogen recognition signaling in chronic infection can help skew and sustain dysregulated host inflammatory responses over a much longer time frame. Our host cells possess a range of pattern recognition receptors (PRRs), which equip them to recognize and respond to conserved microbial pathogen-associated molecular patterns (PAMPs) as well as host cell-derived damage-associated molecular patterns (DAMPs). Patterns of PRR expression vary between cell types. Airway epithelial cells, for example, express several toll-like receptors (TLRs), including cell-surface TLR2, TLR4, and TLR5, which can recognize *P. aeruginosa* lipoproteins, LPS, and flagellin, respectively, as well as endosomal TLR9, which can recognize *P. aeruginosa* unmethylated CpG DNA (74–76). It is important to note that no single TLR deficiency significantly compromises the immune response to *P. aeruginosa*, although mice deficient in multiple TLRs do experience enhanced susceptibility to infection (77, 78). TLR activation results in signal transduction through MyD88 and TRIF. MyD88 signaling leads to liberation of NFĸB, as well as assembly of AP-1 downstream of the mitogen-activated protein (MAP) kinase pathways, ultimately resulting in transcription of key cytokines and chemokines which coordinate the host inflammatory response (76). Mice selectively deficient for MyD88 in lung epithelial cells were more susceptible to acute *P. aeruginosa* infection, with reduced recruitment of neutrophils and impaired early bacterial control post-infection (79). MyD88 was also required to control chronic *P. aeruginosa* infection in a murine model, where complete deletion of MyD88 resulted in severe and lethal lung pathology following infection (80, 81). TLR signal transduction can also occur independent of the MyD88 pathway via TRIF-dependent mechanisms which have also been shown to be important for host defense against *P. aeruginosa* infection (82, 83). TRIF activation of IRF3/7 leading to IFN-β production is commonly implicated in antiviral protection, but IRF3/7 have also shown importance in protection against acute lung *P. aeruginosa* infection in mice, where IRF3-deficient mice had reduced lung bacterial clearance, lower neutrophil recruitment, and altered cytokine profiles (84).

TLR activation in both immune and non-immune cells shapes the immune response to *P. aeruginosa* in various ways. TLR activation induces airway epithelial cells to secrete crucial antimicrobial molecules into the mucus layer, including β-defensins, lysozyme, lactoferrin, cathelidicin LL-37, and surfactant proteins A and D (85). TLR signaling can also directly potentiate innate immune cell functions such as phagocytosis and degranulation. Perhaps most importantly, TLR activation results in the production of key pro-inflammatory cytokines such as TNF, IL-6, pro-IL-1β, pro-IL-18, IL-12, and IL-23; as well as chemokines such as CXCL8, CXCL1, CXCL2, CXCL10, CCL2, and CCL5 (86). These molecules coordinate the recruitment and activation of key immune cell populations. In chronic infection, sustained elevated levels of these cytokines are associated with worsened lung function, demonstrating that a robust unresolving inflammatory response to *P. aeruginosa* is both unable to clear infection and drives lung damage, as will be discussed further below.

#### 3.1.2 Inflammasome signaling

In addition to TLR signaling, inflammasome assembly downstream of nucleotide oligomerization domain (NOD)-like receptors (NLRs) also plays an important role in the response to *P. aeruginosa* infection, particularly in chronic lung inflammatory conditions like CF. There are various inflammasomes, but the two most well-characterized in *P. aeruginosa* infection are the NLRP3 inflammasome, which assembles in response to oxidative stress-induced PAMPs and DAMPs, and the NLRC4 inflammasome, which assembles in response to bacterial PAMPs such as the T3SS and flagellin (87–89).

Inflammasome assembly in the cytoplasm leads to the activation of caspase-1 which cleaves pro-IL-1β and pro-IL-18 produced downstream of NFĸB to generate mature IL-1β and IL-18 (90). Inflammasome assembly can also lead to a form of inflammatory cell death termed pyroptosis, which contributes to *P. aeruginosa* killing by macrophages and neutrophils, but can also cause pathological inflammation if overactivated (91, 92). Innate immune cells such as alveolar macrophages and neutrophils are important producers of IL-1β via inflammasome assembly in response to *P. aeruginosa* infection, with airway epithelial cells implicated as playing a lesser role (93). IL-1β is an important cytokine in both acute and chronic *P. aeruginosa* infection, where it has been associated with successful control of infection in acute settings, and deleterious pathology in chronic settings (88, 94–96). High IL-1β levels in sputum produced by pwCF have been associated with *P. aeruginosa* colonization and worsened lung function (97, 98). In the early stages of infection, IL-1β produced by macrophages binds IL-1R on airway epithelial cells and stimulates them to produce neutrophil-recruiting chemokines such as CXCL8, a cascade which can be amplified with chronic infection. Later in infection, IL-1β signaling also triggers the activation of IL-17A secreting T helper 17 (Th17) cells, modulating the Th17/T regulatory cell (Treg) balance, which will be discussed in greater detail below (99). Some studies have reported that the NLRP3 inflammasome is the primary contributor to IL-1β and IL-18-dependent inflammation in *P. aeruginosa* infection and CF, with the NLRC4 inflammasome playing a lesser or even counter-regulatory role (95, 100). Other reports have instead suggested that chronic *P. aeruginosa* isolates from CF are less able to activate inflammasome signaling and that this may be a mechanism of immune evasion that promotes bacterial persistence (101, 102). This stands in contrast to studies which have reported benefits of blocking inflammasome activation in both acute and chronic *P. aeruginosa* and CF (92, 95, 96), indicating that the timing and relative contribution of inflammasome signaling to chronic *P. aeruginosa* infection within and outside the context of CF remains to be fully understood.

#### 3.1.3 PRR signaling in chronic infection

As described above, PRR signaling pathways drive production of pro-inflammatory cytokines and other antimicrobial mediators, as well as recruitment of immune cells to infection sites. The activity of these pathways typically promotes effective clearance of *P. aeruginosa* in acute infection –but what about chronic infection?

As briefly noted above, some studies have reported PRR signaling defects in contexts such as CF which may make it easier for *P. aeruginosa* to gain a foothold in the lungs of these patients, such as defects in the ability of CF airway epithelial cells to respond to pathogens due to reduced cell surface expression of TLR4 (103, 104). Whether these defects are an intrinsic consequence of CFTR mutation, or an adaptive or tolerogenic response to chronic airway inflammation in pwCF, is a topic that remains controversial. Similar findings have been reported for other chronic lung conditions like COPD, where airway epithelial cell TLR4 expression has been shown to be downregulated by cigarette smoke exposure (105).

*P. aeruginosa* may also have mechanisms to exploit PRR signaling pathways to improve bacterial persistence. For example, TLR5-mediated recognition of flagellin has been reported in pwCF to mediate the formation of granulocytic myeloid-derived suppressor cells (MDSCs). These MDSCs are capable of suppressing Th17 responses, discussed in more detail below, which may indirectly limit neutrophil activation and recruitment. This suggests that flagellated *P. aeruginosa* may be able to exploit TLR5 signaling to suppress antibacterial immune responses which may be relevant for the establishment of chronic infection (106). Interestingly, in the patients profiled in this study, the presence of MDSCs actually correlated with improved lung function, which suggests some benefit of these anti-inflammatory MDSCs in controlling chronic inflammation. However, these patients primarily possessed flagellated nonmucoid *P. aeruginosa* isolates, in contrast to the mucoid non-flagellated isolates more common in adult pwCF with established disease. This suggests that targeting TLR5 signaling may be useful for *P. aeruginosa* in initially establishing chronic infection, but no longer beneficial once the bacteria have established chronicity and are able to transition to a nonmotile, biofilm-living phenotype.

Once *P. aeruginosa* establishes chronic infection, it undergoes adaptations which render it less susceptible to recognition by PRRs, leading to immune evasion which favors persistence. Chronic *P. aeruginosa* isolates often undergo LPS modifications such as loss of O-antigen and lipid A modifications, and downregulate expression of flagella, pili, and T3SS which prevent TLR5 and NLRC4 inflammasome-mediated recognition (107). Encasement in a biofilm matrix may also hide PAMPs from PRRs, and bacteria in lung biofilm communities have been visualized to sit on top of airway epithelial cells rather than in contact with them like planktonic bacteria, further reducing contacts and chances for recognition (22, 108). However, despite these immune evasive adaptations, the inflammatory response to chronic *P. aeruginosa* infection is robust and characterized by extensive release of DAMPs in addition to PAMPs which drive vicious cycles of inflammatory mediator production and immune cell recruitment, which will be detailed below.

### 3.2 Non-immune cells

The epithelial lining of the lung is comprised of diverse cell types whose composition varies between the upper and lower respiratory tract. The trachea, bronchi, and bronchioles are lined by a pseudostratified epithelium dominated by ciliated cells, with occasional secretory cells including club, neuroendocrine, and goblet cells, as well as basal progenitor cells located below the epithelium (85). The tight junctions between airway epithelial cells provide a critical mechanical barrier which protects the lung parenchyma from the contents of the airway lumen (85). As previously mentioned, the luminal surface of the airway epithelium is coated in a thin layer of pericellular fluid which allows for effective ciliary beating, with a layer of protective mucus on top that entraps particles and pathogens to be removed by this mucociliary elevator (85). This mucus barrier is maintained by fluids, mucins, and host defense proteins produced by the epithelial and secretory cells of the airway epithelium. Disruptions to normal mucociliary clearance, as described above for conditions like CF, NCFB, and COPD render the lung susceptible to colonization by opportunistic pathogens like *P. aeruginosa*.

Mucociliary clearance is perhaps underrecognized as one of the principal structural immune defense mechanisms in the lung. Modelling mucociliary clearance is still a difficult task, though numerous strategies have been developed (109–111). However, it appears that there are still important gaps in effectively modelling mucociliary clearance in disease contexts such as CF, COPD, and NCFB, as well as modelling the mechanistic effects of infection with pathogens such as *P. aeruginosa* and biofilm formation on already-impaired mucociliary clearance in these conditions (109). Work has been done to demonstrate that the composition and concentration of airway mucus in CF influences the biophysical properties of *P. aeruginosa* biofilms, making them more robust and able to withstand physical and chemical challenges (112). From a therapeutic point of view, impaired mucociliary clearance and mucus accumulation in diseases such as CF limit the penetration and functionality of anti-*P. aeruginosa* antibiotics (113, 114), and targeting mucus specifically with mucolytic therapies has been shown to both improve the function of antibiotics like tobramycin and improve mucociliary transport (115). It has long been recognized that mucociliary clearance is an important readout for the efficacy of CF therapies (116), as we are now seeing with new highly effective CFTR modulator therapies (HEMT). Even partially restoring mucociliary clearance with HEMT is significantly impacting the landscape of lung infections in pwCF (117), as will be discussed further in section 4.4. Overall, the relationship between infection and mucociliary clearance warrants further exploration from a mechanistic point of view.

Moving from structural barriers to specific cell types, airway epithelial cells themselves play crucial roles in initiating and propagating the immune response to *P. aeruginosa* infection. As described above, PRR-mediated recognition of *P. aeruginosa* stimulates airway epithelial cells to produce cytokines and chemokines such as TNF, IL-6, IL-1β, CXCL8, CCL2, CCL5, and CXCL1 which lead to significant recruitment and activation of neutrophils, monocytes, and other immune cell populations. Airway epithelial cells also respond to cytokine production by other cell types, such as IL-1β production by alveolar macrophages, furthering cycles of inflammatory cytokine production which, in acute infection, resolve with pathogen clearance and the ensuing clean-up, but in chronic infection, perpetuate as a consequence of unresolved pathogen and damage-associated signals.

Airway epithelial cells also undergo remodeling in response to chronic infection as they are battered by excessive inflammation. They undergo significant bystander damage from neutrophil products such as reactive oxygen species (ROS) and neutrophil elastase (NE), which will be detailed below. Destruction of lung epithelium results in release of danger signals and leakage of profibrotic mediators such as growth factors and coagulation components which activate underlying fibroblasts to drive lung fibrosis (118–120). In these ways, continual lung injury over time results in disrupted homeostatic responses and tissue repair mechanisms, leading to irreversible lung damage and functional decline.

### 3.3 Innate immune cells

#### 3.3.1 Macrophages

Macrophages are the primary innate immune population resident within the lung. The lung is populated by two main macrophage populations at steady-state: alveolar macrophages located in the lumen of the airways, and interstitial macrophages found in the lung parenchyma. During infection, monocytes are recruited from the circulation in large numbers and polarized by the lung inflammatory milieu to differentiate into different macrophage subsets, which exist on a continuum from the prototypical inflammatory “M1” macrophages to anti-inflammatory/tissue-repair/regulatory “M2” macrophages (121). These macrophage populations play numerous key roles in infection, including phagocytosis of pathogens and debris; production of cytokines to shape the immune milieu; and tissue remodeling and repair.

Amongst the first innate immune cells to respond to *P. aeruginosa* infection in the lung are tissue-resident alveolar macrophages, which can begin to mount anti-pathogen responses in the first few hours following infection. Alveolar macrophages are a unique population of macrophages that have been described as the “sentinels” of the lung. Under healthy conditions, they are responsible for the maintenance of homeostasis through phagocytosis of dead cells and debris to help clear inflammatory stimuli from the lung, protecting the thin alveolar barrier where gas exchange takes place (122, 123). Alveolar macrophages also play an important role in surfactant homeostasis (121). Dysregulated surfactant homeostasis contributes to various chronic lung diseases, including CF, where it is associated with increased susceptibility to infection (124, 125). In infection, once alveolar macrophages are activated by pathogens and cytokine signals from nearby epithelial cells, they release a network of cytokines and chemokines that contribute to the recruitment and activation of neutrophils, monocytes, dendritic cells, natural killer (NK) cells, and later T cells within sites of infection (126, 127). Alveolar macrophages can also directly phagocytose and kill *P. aeruginosa*, which will be described in greater detail below. While neutrophils are the major cell type responsible for driving pathology in *P. aeruginosa* infection, as will be detailed below, alveolar macrophages play crucial roles in regulating neutrophil lifespan, and phagocytosing dying neutrophils to limit neutrophil-induced tissue damage (128). Dysregulations in the ability of alveolar macrophages to regulate neutrophil homeostasis during chronic infection can drive further pathology.

The specific role of interstitial macrophages in chronic *P. aeruginosa* is less well-characterized, as they are a highly heterogenous population originating from various sources (121). Interstitial macrophages have only recently been discovered to participate in lung fibrosis (129), which may be important at late stages of chronic infection and chronic lung disease.

During chronic infection, monocyte-derived macrophages are continually recruited from the circulation in a CCR2-dependent manner, as tissue-resident alveolar macrophages are diminished, gradually replacing both alveolar and interstitial macrophages over time (130). This population has been shown to contribute to excessive neutrophil recruitment through CXCL2 production and drive detrimental tissue remodeling and fibrosis through production of high levels of TGF-β (130). Use of CCR2 inhibitors to block recruitment of monocyte-derived macrophages in a chronically inflamed CF model reduced both neutrophilic infiltration and TGF-β levels (130). This emphasizes that better understanding the contribution of specific macrophage subsets to chronic inflammation will allow for more specific targeting of detrimental populations.

The various macrophage populations described above recognize and kill *P. aeruginosa* via several pathways. As mentioned, macrophages express numerous PRRs, including TLRs and NLRs, which allow them to sense and respond to pathogens like *P. aeruginosa* (74). Via these pathways macrophages produce a wide range of cytokines that shape the response to infection, allowing for the recruitment of neutrophils and later other immune cells that are instrumental to bacterial control (126, 131). Airway macrophages are an important source of numerous pro-inflammatory cytokines and chemokines including IL-1α, IL-1β, IL-6, TNF-α, IFN-γ, IL-18, IL-4, IL-23, CXCL8, CCL2, CCL3, CCL4, and CCL20, amongst others (132–134). Not all macrophages produce the same cytokines in the same frequencies. Macrophages are polarized by cytokine signals received from airway epithelial cells to amplify specific cytokine responses (122, 135). The cytokine-producing arm of macrophages has been shown to be essential for protection in various murine *P. aeruginosa* infection models. Macrophage cytokine production has been correlated with effective neutrophil recruitment in acute infection (134, 136). Case studies in pwCF and murine models of chronic *P. aeruginosa* infection have shown that this same cytokine production and ability to recruit other immune cells can be detrimental, showcasing that responses that are protective early in infection can become pathogenic later (121, 130, 137). In acute infection, this recruitment of immune cells leads to effective bacterial clearance and infection resolution, whereas in chronic infection, recruited immune cells are unable to clear infection and instead perpetuate inflammatory signaling and damage within the lung.

One of the most important functions of macrophages is phagocytosis. Phagocytic killing is crucial for the control of *P. aeruginosa*. Individuals with genetic deficiencies in phagocyte responses are predisposed to *P. aeruginosa* infection (138), and depletion of phagocytes in animal models increases susceptibility to and lethality of infection (139). Once bacteria such as *P. aeruginosa* are phagocytosed, they are killed intracellularly within phagolysosomes by NADPH oxidase-dependent ROS production (140). Like many Gram-negative pathogens, *P. aeruginosa* is susceptible to opsonic phagocytosis. Briefly, phagocytes such as macrophages express antibody-binding Fc receptors and complement binding receptors which allow them to recognize opsonized extracellular *P. aeruginosa* (141). Other proteins such as surfactant A and C are reported to bind *P. aeruginosa* and assist in this process (142, 143). However, alveolar macrophages tend to express relatively low levels of opsonic receptors, suggesting that this may not be their primary pathway of *P. aeruginosa* clearance in lung infections (144). Macrophages are also capable of killing *P. aeruginosa* by non-opsonic phagocytosis, which may play an important role in controlling bacteria at early stages of colonization (145). Non-opsonic phagocytosis can be mediated by a variety of different receptors (144, 146). One key element that macrophages and other phagocytes use to recognize *P. aeruginosa* is flagella, both via TLR5-mediated recognition of flagellin as well as recognition of specifically swimming motility itself (145, 147, 148). While flagella expression is important for initial invasion and colonization of the lung, likely via binding to host cell Muc1 mucin in the upper airway (149, 150), loss of flagella is a common occurrence in chronic *P. aeruginosa* infection as bacteria transition to a nonmotile biofilm lifestyle. Loss of flagella in chronic *P. aeruginosa* isolates has been shown to increase bacterial resistance to phagocytosis by over a hundred-fold (145, 151). In addition, shifting of *P. aeruginosa* to the mucoid phenotype, a common adaptation in chronic infections, also prevents phagocytosis (152–155). Alginate production by mucoid *P. aeruginosa* has been shown to inhibit receptor-ligand interactions important for phagocytosis in human and murine macrophages (156). *P. aeruginosa* strains overexpressing the exopolysaccharide Psl limit complement deposition by preventing access to the bacterial outer membrane (155). Persister cells, a metabolically dormant subpopulation of antibiotic-tolerant bacteria prevalent in chronic infection, also resist C5b complement deposition, are engulfed at a ten-to-one-hundred-fold lower rate following opsonization, and even resist killing once engulfed (157). These various adaptations of chronic *P. aeruginosa* to avoid and modulate macrophage phagocytosis indicate that this may represent an initially effective mechanism for early control of infection, suggesting that improving the ability of macrophages to phagocytose and clear *P. aeruginosa* in chronic infections could be a therapeutic avenue.

One additional confounder when considering the role of macrophages in chronic *P. aeruginosa* infection within the specific context of CF, is the controversy over whether CFTR defects result in intrinsic macrophage defects that predispose these patients to infection. Early studies reported that CF macrophages had phagocytic defects, and once CFTR expression was identified in macrophages, studies suggested that loss of CFTR resulted in defects in their phagolysosome acidification (158–161). However, these findings have been contradicted by recent studies demonstrating that various populations of CF macrophages do not have defects in phagocytosis or phagolysosomal acidification, as well as results suggesting that macrophage defects in CF are instead a consequence of chronic inflammation (162, 163). Even more recent work in pwCF receiving HEMT have reported that CFTR modulators are able to potentiate CF macrophage function, restoring their ability to phagocytose and effectively kill *P. aeruginosa* as well as altering their inflammatory profiles (164–166). Whether this indicates that CFTR modulators are acting directly on intrinsic CFTR-mediated defects in macrophages or are mediating improved macrophage function by reducing chronic lung inflammation is still unclear. A recent study demonstrated that CFTR modulators were in fact able to enhance phagocytosis in both CF and non-CF macrophages, suggesting that the previously reported ability of CFTR modulators to improve macrophage function may not be CF-specific (167). Better understanding how macrophage function is modulated in the context of chronic *P. aeruginosa*-infection-associated inflammation will be important for the development and rational use of novel therapies.

#### 3.3.2 Neutrophils

Neutrophils are the earliest circulating cells to respond upon *P. aeruginosa* infection in the airway, able to arrive at sites of infection within minutes to hours, and they play an essential role in controlling acute infection (168). Neutropenic individuals are far more susceptible to *P. aeruginosa* infection, and neutrophil depletion in mice renders them lethally susceptible to extremely low inocula of *P. aeruginosa* (138, 168, 169), underscoring their importance. Neutrophils have also been highlighted as central players in the pathology of chronic lung diseases including CF (170) and COPD (171, 172). The interplay between neutrophil dysregulation in these diseases and *P. aeruginosa* susceptibility is, as will be discussed below, complex and likely bidirectional, with disease-specific neutrophil impairment leading to enhanced *P. aeruginosa* susceptibility, and *P. aeruginosa* infection enhancing neutrophil-mediated lung damage and disease progression.

Neutrophils are recruited to sites of infection by inflammatory cytokines, particularly the chemokine CXCL8 [murine homologues are keratinocyte chemoattractant (KC) and macrophage-inflammatory protein 2 (MIP-2)], which binds CXCR1 and CXCR2 receptors on neutrophils (173–175). Neutrophil transmigration from circulation into the lung also requires the CD18 integrins, loss of which increases susceptibility to acute *P. aeruginosa* pneumonia (176). Other soluble mediators important for the migration and activation of neutrophils include cytokines TNF and IL-1β, complement anaphylatoxin C5a, and lipid mediators like leukotriene B4 (LTB4) (170). IL-17 cytokines, namely IL-17A, also play an important indirect role in neutrophil accumulation and activation by inducing local airway epithelial cells to produce more neutrophil mobilizing factors (177, 178).

At sites of infection, neutrophils are activated by a variety of signals, including recognition of bacterial PAMPs by PRRs, as previously mentioned; cytokines and chemokines, such as CXCL8, which in addition to mediating neutrophil migration, also activates neutrophil effector functions such as degranulation and respiratory burst; and opsonins such as complement proteins and antibodies, which tag bacteria for phagocytosis (179, 180).

Neutrophils can kill *P. aeruginosa* through a variety of mechanisms, including phagocytosis, degranulation, and the production of neutrophil extracellular traps (NETs). Neutrophil phagocytosis occurs via mechanisms like those described above for macrophages. Neutrophils engulf bacteria into phagosomes, which fuse with lysosomes to form phagolysosomes, where NADPH oxidase-generated ROS and antimicrobial granule contents come together to kill internalized bacteria (181). In addition to phagocytic killing, neutrophils release a variety of antimicrobials and ROS into the extracellular environment through the process of degranulation. Neutrophil granule contents which are capable of inhibiting *P. aeruginosa* include antimicrobial peptides, such as α-defensins and cathelicidin LL-37; proteases such as neutrophil elastase (NE) and matrix metalloprotease-9 (MMP-9); other enzymes like myeloperoxidase (MPO) and lysozyme; and other soluble antimicrobials like lactoferrin (182, 183). These products, particularly NE and ROS, have been extensively associated with tissue damage and lung pathology in chronic *P. aeruginosa* infections in CF, COPD, and NCFB (170). Neutrophils can also undergo a unique form of inflammatory cell death termed NETosis where, in response to pathogens such as *P. aeruginosa*, neutrophils condense and extrude their cellular DNA coated in granule-derived antimicrobial proteins, such as NE, to ‘trap’ extracellular bacteria (184, 185). These NETs also play crucial roles in neutrophil-mediated lung pathology in chronic infection, which will be discussed in detail below. In addition to these direct killing mechanisms, neutrophils can also indirectly shape the immune milieu. Though not historically considered major cytokine producers, neutrophils do also produce a range of cytokines and chemokines, such as TNF, IL-1β, IFNγ, IL-4, IL-10, IL-13, IL-17, CXCL8, and CXCL9/10, which contribute to shaping the inflammatory response to infection (178, 186–189). Neutrophils, primarily by virtue of their large numbers, have been identified as major producers of IL-1β in the lung milieu of pwCF, downstream of NLRP3 inflammasome activation (96). Targeting neutrophil-mediated cytokine production in neutrophilic diseases may be another method to offset maladaptive inflammation (96).

Despite the importance of neutrophil-mediated killing mechanisms for clearance of *P. aeruginosa* in acute infection, many of these products have non-specific ‘off-target’ effects on host tissues which, in conditions like chronic infection, prevent effective immune clearance, and ultimately even heighten lung damage and contribute significantly to lung pathology. The neutrophil product NE has been shown to cleave the complement receptor CR1 expressed on neutrophils (190), as well as the opsonin iC3b bound to the surface of *P. aeruginosa* (191). This results in an opsonin-receptor mismatch which impairs the complement-mediated phagocytosis of opsonized *P. aeruginosa*, the stimulation of ROS production, and ultimately the ability of neutrophils to effectively kill opsonized *P. aeruginosa* (191). Potentially as a consequence of this thwarted phagocytosis, and other mechanisms, neutrophilia is a common hallmark of chronic *P. aeruginosa* infection, with neutrophils commonly observed surrounding biofilms in high numbers (170, 192, 193). Biofilm formation is one of several bacterial strategies which allows *P. aeruginosa* to resist many neutrophil killing mechanisms (152, 155). Unfortunately, neutrophils have also been shown to unwittingly promote *P. aeruginosa* biofilm formation. *P. aeruginosa* has been reported to co-opt extracellular DNA produced by NETosis for its biofilm extracellular polymeric substance (EPS) (194). Furthermore, excessive NETosis has been associated with increased mucus viscosity, further hampering mucociliary clearance of bacteria, and ultimately exacerbating lung injury in CF as well as NCFB (185, 195–197). Several reviews provide greater detail on the detrimental roles of NETosis in CF and chronic *P. aeruginosa* infection (170, 198–201). Neutrophils can also become dysregulated by chronic inflammatory contexts. For example, neutrophils isolated from pwCF chronically infected with *P. aeruginosa* exhibit prolonged survival (202–204). Neutrophil apoptosis is a tightly regulated process which limits the release of neutrophil contents to prevent damage to the surrounding tissue. Apoptosis is coordinated with non-inflammatory clearance (efferocytosis) by phagocytes to successfully resolve inflammatory responses (205). In COPD, cigarette smoke has been shown to impar the ability of alveolar macrophages to effectively phagocytose apoptotic neutrophils (206). In conditions of chronic infection and continuous inflammatory signaling, it is likely that the dysregulation of neutrophil activation and survival leads to a viciously intensifying inflammatory cycle that is unable to resolve. This central role of neutrophils in coordinating the maladaptive response to pathogens such as *P. aeruginosa* in chronic lung disease has led to significant efforts to therapeutically modulate neutrophil activity and products, which will be discussed in a later section.

#### 3.3.3 Other innate immune cells

Neutrophils and macrophages represent the main innate immune populations involved in the response to *P. aeruginosa* in the lung. However, there are other immune cells which play supporting roles, or whose roles in *P. aeruginosa* infection, particularly chronic infection, are still being characterized.

Dendritic cells (DCs) are the professional antigen-presenting cells of the immune system, functioning to bridge the innate and adaptive responses by activating and differentiating naïve T cells into effector subsets. This has relevance for chronic *P. aeruginosa* infection as T cell polarization toward Th2 and Th17 immunity has been associated with increased pathology and worsened outcomes, for distinct reasons which will be discussed further below (207–209). Limited work has investigated the roles of DCs in chronic *P. aeruginosa* infection, but they are known to be activated and express co-stimulatory molecules CD80 and CD86 in the lung and regional lymph nodes in response to *P. aeruginosa* (210). Furthermore, *P. aeruginosa* appears able to modulate DC subpopulations, resulting in a skewing of the resting conventional DC (cDC) population toward cDC2s that support a Th2 response rather than cDC1s that promote Th1 and bacterial clearance (211). This fits into the existing paradigm of Th2-skewed immunity in chronic *P. aeruginosa* and suggests that DCs may be contributing to this later T cell imbalance. *P. aeruginosa* quorum sensing molecules have also been shown to impair the ability of DCs to induce T cell proliferation, which could also impair the generation of an effective adaptive response (212).

Other granulocytic cell populations, namely mast cells and eosinophils, play important roles in some chronic airway conditions, such as asthma and COPD, and as such have also been investigated for potential roles in CF and chronic lung inflammation associated with *P. aeruginosa* (213, 214). Eosinophils were found to be activated and contribute to lung destruction in CF, though they play a far more minor role than neutrophils owing at least in part to their much smaller population (215). In bronchiectasis, eosinophilic inflammation was associated with *P. aeruginosa* infection (216). Eosinophils have been recognized to play an important role in a subtype of COPD (217, 218), although eosinophilic COPD appears less-associated with *P. aeruginosa* infection than neutrophilic COPD (48). Overall, eosinophils appear to play only a minor role in chronic *P. aeruginosa* infection in the context of CF or other diseases. Mast cells also respond to *P. aeruginosa*, and may contribute to the inflammatory cascade by producing cytokines and chemokines such as CXCL8 and CCL20 to further recruit neutrophils and monocyte-derived macrophages to the respiratory tract (219–221). However, the involvement of these cells is also understudied.

Outside the myeloid compartment, various innate lymphoid populations have also been implicated in the response to *P. aeruginosa* lung infection. Natural killer (NK) cells are most commonly associated with protection against viral infection and cancer but have also been reported to kill some bacteria (222). NK cells can be recovered from bronchoalveolar lavage fluid (BALF) following *P. aeruginosa* lung infection, suggesting recruitment in response to infection, and NK cell depletion has resulted in increased mortality and *P. aeruginosa* lung bacterial burden in acute murine models (222, 223). NK cells have recently been demonstrated to directly kill extracellular *P. aeruginosa* through release of granzymes B and H which damage the bacterial membrane and induce ROS production resulting in bacterial death (224). Work remains to be done to assess whether NK cells play a meaningful role in chronic *P. aeruginosa* infection and whether they could be therapeutically harnessed.

Innate lymphoid cells (ILCs) are a plastic group of innate lymphocytes that reside in tissues, particularly at mucosal surfaces, where they function primarily as cytokine secretors (225). ILCs are divided into subsets that mirror conventional T helper cell subsets, with ILC1s producing Th1 cytokines, ILC2s mirroring Th2s, and ILC3s generating Th17 cytokines (225). ILCs play a broad spectrum of roles, from protecting against pathogenic threats including viruses, bacteria, and helminths; regulating microbiota crosstalk; promoting allergic and autoinflammatory reactions; and mediating tissue maintenance and repair (226). ILC2s are the predominant ILC subtype found in the lung; however, it is ILC3s, with their ability to produce IL-17 and IL-22, which have been implicated in the response to lung *P. aeruginosa* infection (227). One study found that ILC3s were responsible for the majority of innate immune cell production of IL-17 in the lungs in response to *P. aeruginosa* infection, which in this model was identified as critical in preventing the transition to established chronic infection (228). Another study utilizing *Rag*/γ-chain^-/-^ mice deficient in T cells, NK cells, and ILCs had an increased *P. aeruginosa* load compared to classical *Rag^-/-^* knockout mice (lacking only T and B cells), suggesting a potential contribution of innate lymphocytes to infection control (229). However, the contributions of ILCs to *P. aeruginosa* infection and chronic lung disease remain understudied.

Unconventional immune cell populations, such as γδ T cells and natural killer T (NKT) cells, have also been associated with *P. aeruginosa* infection. γδ T cells are a rare population of innate-like T cells which are resident in tissues and mucosal sites, including the lung (230). γδ T cells have been implicated in the response to acute *P. aeruginosa* infection, as γδ T cell-deficient mice exhibited enhanced susceptibility to intranasal *P. aeruginosa* challenge characterized by increased bacterial load, reduced survival, delayed neutrophil recruitment, and enhanced production of specific pro-inflammatory cytokines IL-1β, IL-6, and TNF (231). This effect did not appear to be mediated primarily by IL-17, as γδ T cells were not major IL-17 producers in this model (231). This stands in contrast to other reports which found that IL-17 production by γδ T cells was important for neutrophil chemotaxis and elimination of *P. aeruginosa* during acute murine lung infection, as well as for B cell stimulation resulting in antibody production (232, 233). Extrapolating these results to chronic infection, γδ T cells may play a role in modulating the cytokine milieu of the inflamed airway, potentially to detrimental effect. Another unconventional T cell subset which straddles the border of innate and adaptive immunity is the NKT cell. These cells recognize lipid and glycolipid antigens rather than peptides. Reports on the role of NKT cells in *P. aeruginosa* infection are conflicting and appear to differ based on experimental conditions including *P. aeruginosa* strain and mouse background. *Jα18^-/-^* mice deficient in NKT cells were reported to have impaired clearance of *P. aeruginosa* strain D4, but not PAO1 or PAK (231, 234, 235). These conflicting results may depend, in part, on host genetic background, and differing levels of NKT cells. Indeed, one study found that NKT cells played an important role in *P. aeruginosa* clearance from the lungs of BALB/c mice, which have greater NKT cell frequencies, but had no significant role in C57BL/6 mice (236). This strain-dependent difference was also associated with different NKT cell cytokine profiles as well as differential impacts on neutrophil recruitment (236), again highlighting the importance of specific cytokine production and modulation of neutrophils in shaping immune responses to *P. aeruginosa*. The roles of γδ T cells and NKT cells in human *P. aeruginosa* infection and CF are not known. However, NKT cell activation does not appear to be impaired by CFTR dysfunction in pwCF (237).

### 3.4 Adaptive immunity

The adaptive immune system also plays crucial roles in *P. aeruginosa* infection, particularly in chronic infection. Early work established the relevance of adaptive immunity to *P. aeruginosa* infection by demonstrating that *Rag2* ^-/-^ mice, which are devoid of classical T and B cells, had an impaired ability to clear *P. aeruginosa* from the lungs, resulting in decreased survival (235). The ability of B cells to respond with robust antibody production in chronic *P. aeruginosa* infection has been long-established; however, the reasons behind the limited protective ability of this response remain to be fully elucidated. T cell responses, particularly CD4+ T cell responses, have also been shown to play critical roles in shaping the cytokine milieu in chronic *P. aeruginosa* infection and chronic lung disease more broadly. Untangling the relationship between adaptive immune responses and chronic *P. aeruginosa* infection has proven somewhat challenging, as experimental models of chronic infection have various limitations. It remains difficult to establish consistent and long-lasting chronic infections in murine models (238, 239), which makes it difficult to study adaptive populations that require at least a week to mount a primary response.

#### 3.4.1 T cells

Various T cell subsets, specifically CD4+ T helper (Th) subsets, have been implicated in the pathology of chronic *P. aeruginosa* infection, particularly in the context of CF. Several early studies implicated Th2 skewing as a major component of the maladaptive inflammatory response and susceptibility to pathogens such as *P. aeruginosa* which occur in pwCF (207, 240). Later, with the discovery of Th17 cells, this paradigm was broadened to implicate both populations in shaping inappropriate responses to *P. aeruginosa* infection in chronic lung disease (209).

Th2 cells are polarized in the presence of IL-4 and upregulate the transcription factor GATA-3. Th2 cells produce the cytokines IL-4, IL-5, and IL-13 (241). Th2 immunity is associated with combatting extracellular pathogens, particularly parasites, but also contributes to allergic airway diseases such as asthma (242). Early studies demonstrated that a Th2-dominated immune response was associated with chronic *P. aeruginosa* infection in pwCF compared to uncolonized pwCF (207, 240). Even outside the specific context of CF, chronic *P. aeruginosa* infection was more severe in Th2-skewed BALB/c mice than Th1-skewed C3H/HeN mice (243). High production of prototypical Th1 cytokines, namely IFNγ, correlated with improved lung function in pwCF, and resistance to *P. aeruginosa* re-infection in murine models is associated with a shift toward a Th1-dominant response (208). Given these observations, the question becomes: what factors are influencing this skewing of T helper cell subsets? Some work has implicated bacterial products as playing a direct role in skewing T cell immunity. For example, N-acyl homoserine lactone quorum sensing (QS) molecules produced by *P. aeruginosa* have been shown to suppress Th1 cytokines and proliferation, skewing the balance toward Th2 cytokines and Th2 immunity (212, 244). Recently, the *P. aeruginosa* protease elastase B (LasB) was reported to induce Th2-associated genes through activation of epithelial growth factor family members (245). But this representation of a Th2 vs. Th1 skewing is an oversimplification which fails to account for the heterogeneity of cytokine responses and other immune polarizations, particularly the contribution of Th17 immunity, which has more recently been described to also play an important role in chronic *P. aeruginosa* lung infection.

Th17 cells are polarized in response to TGF-β, IL-6, IL-21, and IL-23, and defined by the transcription factor RORγt (246). Th17 cells produce IL-17 family cytokines, primarily IL-17A, which is a proinflammatory cytokine involved in granulopoesis, as well as recruitment and activation of neutrophils. IL-17A stimulates the production of several other proinflammatory cytokines and chemokines, namely TNF, IL-6, GM-CSF, and CXCL8 (247, 248). Th17 responses are associated with protection from extracellular pathogens such as bacteria; however, they can also lead to inflammation and autoimmunity. Studies have reported that Th17-induced cytokines are heightened in sputum of *P. aeruginosa*-colonized pwCF, and that levels of IL-17A are also increased in pwCF undergoing exacerbations (209, 249, 250). Heightened levels of Th2 and Th17 cytokines also correlated with subsequent *P. aeruginosa* infection in pwCF, suggesting that Th2-Th17 immunity may be a risk factor for (re-) infection as well as a potential marker of ongoing infection (249). However, IL-17 may not play a solely detrimental role. In a murine model of chronic *P. aeruginosa* lung infection, IL-17 was reported to be required for infection control (228). In this model, loss of IL-17RA increased 2-week infection rates from 25% in wild-type (WT) animals to 100% in IL-17RA^-/-^ mice and increased overall bacterial loads. However, this model surprisingly noted no significant differences in neutrophilic infiltration with loss of IL-17RA. By contrast, in a mouse model of COPD and *P. aeruginosa* infection, treatment with neutralizing antibodies targeting IL-17A significantly reduced *P. aeruginosa* bacterial burden, induced significantly less neutrophil infiltration, and improved lung function (251). Administration of exogenous IL-17A instead exacerbated *P. aeruginosa*-mediated inflammation and lung dysfunction. The differences between these two models may stem from differences in basic infection context: healthy mice as compared to mice with existing chronic inflammatory lung disease. In the latter case, IL-17 may mediate pathologic responses by amplifying a harmful inflammatory cascade, rather than initiating a protective response in the short-term leading to infection resolution, as has been suggested by others (228). Taken together, this work suggests that IL-17 may play a protective role in preventing the transition from acute or intermittent infection to chronic infection by mediating a protective neutrophilic response; however, once chronic infection has been established, robust production of IL-17, primarily mediated by Th17 cells, plays a detrimental role in driving lung pathology and a non-resolving immune response.

Other considerations implicated in maladaptive T cell immunity to *P. aeruginosa* include the reported impairment of regulatory T cell (Treg) function in chronic *P. aeruginosa* infection. Some studies show this occurs independently of CF (252). Cross-regulation between Th17 and Treg lineage commitment does imply that a Th17-skewed immune response would negatively regulate inducible Tregs, leading to a lessened Treg response (253). Other reports suggest that Treg dysfunction is a feature of CF disease which may be affected by *CFTR* mutations, as CFTR potentiator/corrector therapies appear to affect Treg function (254, 255). Most likely, the combination of both factors leads to greater impacts on Tregs, as reported by one study which showed that Tregs from pwCF or *Cftr^-/-^* mice had an impaired ability to suppress conventional T cells, and that this effect was enhanced by *P. aeruginosa* infection. This study also observed that chronic *P. aeruginosa* decreased overall Treg levels in pwCF (256). Loss of Tregs appeared to primarily impact the memory compartment and may prevent rebalancing of the immune response in chronic infection (256).

Several outstanding questions remain regarding the roles of T cell immunity in chronic *P. aeruginosa* infection. Are chronic lung diseases, such as CF, skewed toward Th2/Th17 immunity, and poor Treg responsiveness, prior to and independently of bacterial infection, or does infection with pathogens such as *P. aeruginosa* drive this response? Is a Th17 response protective in acute infection and maladaptive in chronic infection, and if so, how and when does this shift occur? Would improving Treg function allow for better control of dysregulated inflammatory responses to chronic *P. aeruginosa*? These questions, and others, highlight that the T cell response to chronic *P. aeruginosa* infection is complex and still in the process of being untangled.

#### 3.4.2 B cells

B cells respond robustly to chronic *P. aeruginosa* infection through production of high levels of *P. aeruginosa*-specific IgG and IgA antibodies (Abs) and immune complex formation. However, this robust humoral response is not associated with bacterial clearance or clinical improvement and has even in some cases been suggested to be detrimental (257, 258). Though B cell responses were some of the earliest characterized responses to chronic lung *P. aeruginosa* infection (259, 260), they remain poorly understood to this day (257).

Early studies reported *P. aeruginosa*-specific Abs were increased in the presence of the mucoid phenotype and associated with a poor prognosis in CF (261). High serum anti-*P. aeruginosa* Ab levels are associated with persistent infection, to the degree that they have been suggested as a diagnostic tool, indicating that a robust Ab response is not protective or able to clear infection (262, 263). PwCF appear to generate high levels of circulating IgG as well as high levels of primarily IgA serotypes in the ASL lining the lung (257). The formation of immune complexes (ICs) in both sputum and serum has long been reported during chronic *P. aeruginosa* lung infection, primarily in pwCF (259, 264). However, studies have not identified definitively whether IC formation is detrimental. Early work reported that ICs stimulated greater inflammation via complement activation, as well as worse clinical status in patients; however, other longitudinal studies failed to find correlations (265–268). Others have suggested the efficacy of the Ab response to *P. aeruginosa* may be impaired by various factors, including the inflammatory CF lung environment, chronic exposure to *P. aeruginosa* antigens, and phenotypic changes in *P. aeruginosa* including LPS modifications, loss of flagella, overproduction of alginate, and biofilm lifestyle (257). Other factors may include impaired Ab transport in dehydrated ASL and entrapment in thickened biofilms and mucus, as well as increased degradation by host or bacterial proteases which are plentiful in chronically infected lungs (269–271). Several groups have shown anti-*P. aeruginosa* Abs developed in the chronic inflammatory context of CF may have lower avidity or affinity, which negatively impacts their efficacy as opsonins to stimulate phagocytosis, thus preventing bacterial clearance (272–275). A reduced ability of phagocytes to kill opsonized *P. aeruginosa*, both from loss of phagocytic capacity due to chronic inflammatory stimuli or intrinsic defects, as well as evasion methods of *P. aeruginosa*, also limits the functionality of Ab responses. Finally, while Abs may not be able to clear chronic *P. aeruginosa*, they still play an important role by protecting against dissemination from local sites to systemic infection. Overall, B cell responses to *P. aeruginosa* are robust but fail to clear chronic infection, which has implications for development of Ab-based therapies as well as vaccines, discussed in the following section.

## 4 Modulating infection

Historically, treatment of chronic *P. aeruginosa* has relied on antibiotics prescribed upon initial detection of infection (i.e., for eradication) or pulmonary exacerbation in conditions such as CF, COPD, and NCFB. Given the known association between *P. aeruginosa* infection in pwCF and poor outcomes, current clinical guidelines recommend chronic suppressive nebulized antibiotics to control *P. aeruginosa* (276). However, antibiotic use is becoming fraught with the global rise of antimicrobial resistance, which is increasingly being found in *P. aeruginosa* isolates from chronic lung infections (277). Additionally, antibiotics are frequently unsuccessful in clearing *P. aeruginosa* infection in chronic lung diseases, particularly in adults. Once infections transition to a chronic state, they become increasingly resistant to antibiotics through adaptations such as biofilm formation, hypermutability, and the formation of persister subpopulations, as discussed previously. The limited efficacy of both systemic and nebulized antimicrobials indicates a need for novel approaches to both prevent and clear chronic *P. aeruginosa* infections in individuals with chronic lung disease.

However, clearance of infection is not the sole metric by which to reduce pathology and improve outcomes in these patients. As described above, the immune system contributes substantially to lung damage and morbidity associated with chronic *P. aeruginosa* infection, and so represents an additional target to reduce the disease burden of chronic infections in these patients. This section will discuss several key approaches to modulate infection, including methods designed to treat or prevent infection itself by targeting *P. aeruginosa*, immunomodulatory therapies designed to reduce the pathology of infection, and finally therapies designed to correct the innate defects, specifically in CF, which predispose individuals to chronic *P. aeruginosa* infection. Insights from CFTR modulators in terms of changes to immune function and bacterial colonization are beginning to shed new light on the relationship between inflammation and chronic *P. aeruginosa* infection which may have broader applicability in non-CF contexts.

### 4.1 Antibiotics

Two of the earliest antibiotics proven effective for pwCF were inhaled tobramycin and oral azithromycin, which are used in approximately two-thirds of pwCF with chronic *P. aeruginosa* infection, most often in combination (278). These antibiotics are also frequently used to treat *P. aeruginosa* in COPD and NCFB. Tobramycin is an aminoglycoside antibiotic which binds the 30s subunit of the bacterial ribosome to inhibit translation initiation (279). Inhaled long-term use of tobramycin appears to improve lung function and reduce exacerbation rates in patients, likely by helping to keep *P. aeruginosa* under check (280). Azithromycin belongs to the macrolide class of antibiotics and operates by binding to the 50s subunit of the bacterial ribosome and inhibiting translation, although it is largely prescribed in pwCF at non-bactericidal doses for its immunomodulatory function and other mechanisms of action, which will be discussed in section 4.3, rather than anti-*P. aeruginosa* activity (281). Another common antibiotic used in combination with tobramycin is inhaled aztreonam, a beta-lactam antibiotic which inhibits bacterial cell wall synthesis (282, 283). Novel antibiotics as well as combination therapies are still being robustly investigated in CF and other chronic *P. aeruginosa* infection contexts, which have recently been summarized by others (284). Clinical decisions regarding antibiotic treatment in pwCF are beginning to shift in the era of CFTR modulators to account for improvements in lung function, reduced ability to acquire sputum samples, and the changing landscape of bacterial infections (285).

### 4.2 Vaccines

Development of a vaccine to protect against *P. aeruginosa* would represent a significant advance for pwCF and other chronic lung conditions. Vaccine development efforts for *P. aeruginosa* have been ongoing for over half a century but have been hindered by a multiple factors, including the plasticity of *P. aeruginosa* owing in part to its large genome, its diversity of virulence factors and serotypes, the complexity of its pathogenesis, and its interactions with the lung environment and immune system (128). Hundreds of vaccines have been developed pre-clinically, but comparatively few have reached clinical trials (128, 284).

A variety of potential antigenic targets have been investigated using numerous vaccine strategies. LPS has been one of the most well-investigated targets due to its immunogenicity and surface accessibility; however, early vaccines ran into challenges with toxicity, and later vaccines have faced difficulties in ensuring protection against different, mutable *P. aeruginosa* serotypes (286, 287). Flagellar antigens, as well as pili, have also been vaccine targets, given their importance for *P. aeruginosa* virulence and initial colonization. Vaccines solely targeting flagellar antigens elicited some protective antibodies in early-stage clinical trials, but not in sufficient proportions of participants (288). Various outer membrane porins, particularly OprF, have also been investigated as antigenic targets (289). Alginate, an exopolysaccharide which is often overproduced in chronic infections, has also been targeted, with early vaccines inducing the development of opsonophagocytic Abs against alginate which could theoretically potentiate phagocytic responses (290). However, low immunogenicity has led to efforts to enhance the immunogenic properties of alginate, such as conjugation with other antigens to transform it into a T-cell dependent antigen (128). Targeting the T3SS of *P. aeruginosa*, particularly its key component PcrV, has shown some efficacy in reducing airway inflammation and damage in pwCF in a stage II clinical trial (291, 292). Overall, these more recent approaches have transitioned to the use of multiple antigenic targets in combination with various adjuvants to maximize the breadth and types of protection (293–295). Whole-cell killed and live-attenuated bacterial vaccine approaches have been less studied, but several are still under development. Mucosal administration of paraformaldehyde-killed *P. aeruginosa* induced protective immune responses in rat lungs (296). Alternative methods of *P. aeruginosa* inactivation have been recently used, including H_2_O_2_ which was less toxic and resulted in better epitope retention than paraformaldehyde, leading to more effective IgG protection (297). Live-attenuated vaccine strategies have also been effective at inducing multifactorial humoral and cellular immune protection, but some concerns remain about residual virulence (298, 299). Novel vaccine delivery strategies, such as DNA and RNA vaccines, are also being considered against *P. aeruginosa* (289, 300). Various mRNA vaccine candidates are showing promising results, including PcrV and OprF-I vaccines which were recently shown to be able to induce Th1-skewed immunity, confer broad protection, and reduce bacterial load in murine *P. aeruginosa* infection models (289).

Future directions for vaccine development will require a better understanding of what the most effective correlates for immune protection against *P. aeruginosa* are. Humoral immune responses are well-induced by many of these vaccine strategies, but patients with chronic *P. aeruginosa* infection also have robust Ab responses that fail to provide significant protection. Our understanding of T-cell mediated immunity to *P. aeruginosa* is also incomplete: Th2 immunity appears maladaptive in chronic infection, with Th1 responses perhaps being a better target. Th17 immunity may be protective in acute infection but pathogenic in chronic infection, leaving the question of whether it would be an effective correlate of vaccine protection up in the air. Overall, acquiring a greater understanding of immunity to *P. aeruginosa* infection, particularly adaptive immunity, in the context of chronic lung conditions like CF where patients may have inherently dysregulated immune responses, will be critical to developing effective vaccines. Recognizing the variability of protein antigens, as well as their loss during chronic infection –for example, loss of flagella, pili, T3SS—and shielding within biofilms should also be considered when thinking about developing vaccines for populations at risk of chronic *P. aeruginosa* infection. Different routes of administration, such intranasal or inhaled vaccination, may be necessary to induce protection at mucosal sites, which will be important particularly for *P. aeruginosa* lung infections (298, 299). Several reviews have recently been written which go into greater detail on the topic of vaccine development against *P. aeruginosa* (128, 284, 301).

### 4.3 Immunomodulatory therapies

**Figure 4:**
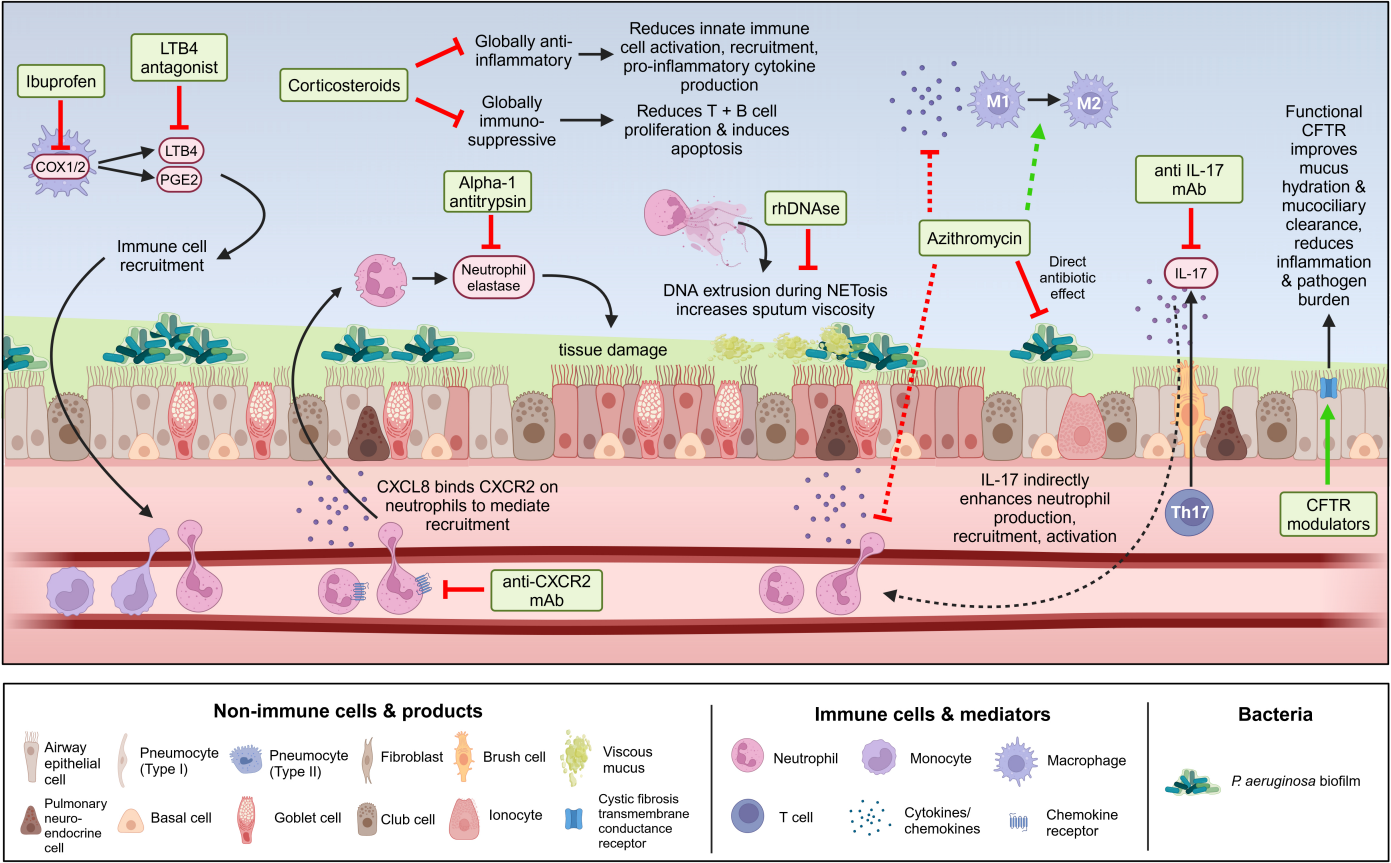
Immunomodulatory therapies available and under development for chronic *P. aeruginosa* infection in the context of chronic lung diseases, particularly CF. Beginning with non-specific therapies, corticosteroids have broad anti-inflammatory, immunosuppressive, anti-proliferative, and vasoconstrictive effects that can reduce the progression of inflammation-driven lung damage in chronic lung disease, but also increase the risk of infections such as *P. aeruginosa*. High-dose ibuprofen acts on the COX-1 and COX-2 receptors to inhibit prostaglandin (ex. PGE2) and leukotriene (ex. LTB4) synthesis, reducing immune cell migration into the airways. Azithromycin is a macrolide antibiotic which, in addition to directly inhibiting bacterial growth, has been reported to have various immunomodulatory functions on innate immune cells like macrophages and neutrophils. Moving into more targeted therapies, alpha-1-antitrypsin is an antiprotease naturally produced in the human lung which restricts neutrophil elastase activity and can be given supplementarily to reduce lung damage. Recombinant human DNAse (rhDNAse) targets enhanced DNA concentrations in sputum because of NET release by neutrophils to reduce sputum viscosity and improve pulmonary function. LTB4 antagonists block the neutrophil chemoattractant and activator LTB4 but caused pulmonary exacerbations in clinical trials. Anti-CXCR2 monoclonal antibodies (mAbs) block the CXCR2 receptor on neutrophils preventing binding of the neutrophil chemoattractant and activator CXCL8. Anti-IL-17 mAbs block IL-17 function to limit neutrophil proliferation and activation. Finally, CFTR modulators, by correcting CFTR dysfunction indirectly modulate the immune system by improving overall lung function, reducing pathogen burden, and reducing inflammation. Figure created in BioRender.com.

Immunomodulatory therapies are intended to target the pathology associated with chronic *P. aeruginosa* infection, rather than treat the infection itself (**Figure 4**). Their importance is highlighted by the significant work reviewed above, which indicates that the immune response to chronic *P. aeruginosa* infection often causes more lung damage and pathology than the infection itself. Neutrophil granule products, reactive oxygen species, and other immune products cause significant direct damage to airway epithelial cells, and inflammatory cytokine production drives further inflammatory processes forward –all of these represent potential targets for immunomodulatory therapies to limit this vicious cycle of inflammation.

The first immunomodulatory therapies used in CF and other chronic lung conditions were broad spectrum anti-inflammatories, such as corticosteroids. Corticosteroid use is generally limited to inhaled and short-term, given the risk of adverse side effects (302), and recent studies have shown they have limited benefits in CF (303). Inhaled corticosteroids have been associated with increased risk of *P. aeruginosa* infection in other chronic lung conditions, such as COPD, due to their broadly immunosuppressive effects (304, 305). Despite this, corticosteroids are still broadly used in COPD (51, 171). By contrast, ibuprofen is a non-steroidal anti-inflammatory drug which has been shown to slow the progression of lung damage in pwCF, associated with its ability to reduce neutrophil migration into the airways by inhibiting leukotriene and prostaglandin production (302). However, uptake in ibuprofen use has been variable in adult CF clinics, potentially limited by concerns regarding side effects (i.e., nephrotoxicity) and the subsequent clinical monitoring associated with long-term high-dose use (302). High-dose ibuprofen is utilized more in pediatric CF patients (306), as they generally have fewer pre-existing comorbidities and additional medications. Azithromycin, described above in the antibiotic section, is notable as it has several postulated mechanisms of action outside of its antibiotic function. The first includes anti-virulence effects, such as suppression of quorum sensing, protein translation, and secretion of virulence effectors (307). Azithromycin has also been reported to have broad immunomodulatory properties. Azithromycin has been shown to reduce neutrophil levels and inflammatory markers such as myeloperoxidase in the serum of pwCF uninfected with *P. aeruginosa* (308) as well as patients with bronchiolitis obliterans syndrome (309), indicating that it may have beneficial immunomodulatory effects even in the absence of chronic *P. aeruginosa* infection. Azithromycin is also used for its immunomodulatory effects in COPD and NCFB (51, 310). Preclinical studies have indicated that azithromycin may inhibit various pro-inflammatory signaling pathways, including NFκB, STAT1, and inflammasome activation, resulting in reduced production of pro-inflammatory cytokines such as TNF and IL-1β and shifted macrophage polarization (311–313). However, other studies have countered the clinical relevance of these findings, reporting that long-term azithromycin use is still primarily effective in pwCF infected with *P. aeruginosa*, suggesting that its immunomodulatory benefits may come second to benefits derived from direct interference with bacterial growth (314–316). Another clinical trial reported that, in addition to azithromycin’s benefits being restricted to the short-term, long-term usage resulted in increased prevalence of macrolide resistance in other organisms (such as *S. aureus*) (317), which raises concerns about its long-term use for non-antibiotic purposes.

Immunomodulatory therapies targeting specific problematic aspects of the inflammatory response to chronic infection are more appealing. Because much chronic *P. aeruginosa* pathology is driven by neutrophils, several strategies have been developed to either target soluble effectors involved in neutrophil recruitment, or neutrophil granule products themselves. Starting with the top of the cascade, antagonists have been developed to block CXCR2, the receptor expressed on neutrophils which binds CXCL8 (amongst other chemokines) to mediate neutrophil migration. Clinical trial results indicated that while use of a CXCR2 antagonist lowered neutrophil numbers in the lungs of pwCF, it had no effect on sputum neutrophil levels, indicating that effects may be localized or that CXCR1 (present in humans but not mice) may compensate (318). Use of a similar CXCR2 antagonist was effective in improving lung function in COPD, indicating that it may be a disease-agnostic treatment (319). Another approach involves neutralizing Abs targeting IL-17, which significantly reduced neutrophil infiltration, lowered proinflammatory cytokine production, and reduced tissue damage in murine lungs during chronic *P. aeruginosa* infection (320, 321). The success of anti-IL-17 Abs like secukinumab in autoimmune diseases have set a precedent for potential translatability (322). Other approaches have been developed to target neutrophil products like NE, which damages the lung epithelium. Alpha 1-antitrypsin (AAT) is an antiprotease naturally produced in the healthy lung to restrain protease activity. In CF, AAT levels are dysregulated, allowing for excess protease activity (323, 324). Hereditary AAT deficiency is also a rare causative agent of COPD (325). Inhaled AAT was found to decrease neutrophilic inflammation and directly impair NE function in *P. aeruginosa* infection without compromising the host’s ability to clear bacteria (326, 327). PwCF treated with inhaled AAT had decreased airway inflammation, measured by parameters including neutrophil infiltration and pro-inflammatory cytokine production (328). Completed trials have not reported increases in lung function, but trials were generally short-term (i.e. up to one month). Studies conducted over longer timeframes may demonstrate the ability of AAT to limit long-term lung function decline. More recently, AAT was demonstrated to directly bind NE embedded in human NETs to limit NET-mediated damage to the epithelial barrier (329). An example of a targeted therapy that has recently entered successful clinical use is recombinant human DNAse (rhDNAse). rhDNAse is currently used to treat pwCF by targeting enhanced DNA concentrations in CF sputum due to NET release by neutrophils. Studies have shown that rhDNAse reduced sputum viscosity, improved lung function, and lowered pulmonary exacerbations; however, clinical trials showed only mild decreases in factors such as NE and CXCL8, with no changes in neutrophils or other inflammatory cytokines in the short term. Long-term treatment over 3 years did prevent increases in several inflammatory mediators, resulting in guideline based recommendations (330–333).

There is a crucial balance to be struck in the use of immunomodulatory therapies in chronic *P. aeruginosa* infection. Successful immunomodulatory therapies must be able to limit inflammation without blunting host immune responses to the degree that the host is vulnerable to new infections or loses the ability to control *P. aeruginosa* infection on the local level, resulting in infectious exacerbation and/or systemic spread. One example of this concern was demonstrated in a phase II clinical trial of a receptor antagonist for LTB4, a potent neutrophil chemoattractant and activator. This trial demonstrated a statistically significant increase in pulmonary exacerbations in patients receiving the LTB4 receptor antagonist amelubant (52-55% in the treatment group versus 33-43% in the placebo), resulting in premature termination of the trial (334). A better understanding of the complex immune processes involved in susceptibility to *P. aeruginosa* infection as well as those involved in lung damage and pathology, is necessary for the careful and rational design of novel immunomodulatory therapies.

### 4.4 CFTR modulators and their effect on *P. aeruginosa* infection in CF

The successful introduction of HEMT to a large proportion of the CF population, estimated to be more than 90% in some countries (335), has dramatically improved both pulmonary and extra-pulmonary health. However, structural lung damage with infections persists.

CFTR modulators fall into 2 functional categories: potentiators, which enhance the channel opening probability of CFTR, and correctors, which improve CFTR folding and trafficking (3, 336). Amongst CFTR modulators, two are considered highly effective. Ivacaftor, targeting the Gly551Asp gating mutation to increase channel time, was the first available HEMT released in 2012 but available only to ~5% of pwCF. Approved in 2019, Elexacaftor-Tezacaftor-Ivacaftor (ETI) is an HEMT relevant to many pwCF given its efficacy for at least one copy of the F508del *CFTR* mutation (and approved by the FDA for other specific rare mutations), accounting for ≥90% of individuals (337). Recent data from a compassionate access program has also demonstrated real-world efficacy across non-F508del mutations.

CFTR modulators have been reported to affect lung inflammation and immune parameters in various ways. Some *in vitro* studies have suggested that CFTR modulators can directly modulate inflammatory responses in *CFTR*-deficient cells. For example, *CFTR^F508del^* epithelial cells treated with lumacaftor/ivacaftor exhibited less exuberant inflammatory responses, decreased production of CXCL8, and enhanced tissue repair following *P. aeruginosa* exposure (338, 339). Animal models have allowed for tracking of the timing of therapy initiation, demonstrating that administration of ivacaftor to *CFTR^G551D^* ferrets *in utero* partially protected animals from multiorgan pathologies caused by CF, and led to reduced mucus accumulation and lung bacterial infections. Withdrawal of therapy at any age led to the rapid onset of pathology including airway inflammation (340). More recently, studies have begun to be published investigating the effects of ETI therapy, though much of the significant work in this area is yet to come. Some studies have reported positive immunomodulatory effects, including significant decreases in blood levels of inflammatory cytokines CXCL8, IL-6, and IL-17A, as well as normalization of peripheral blood immune cell composition (341), even in pwCF with advanced disease (342). However, another study reported no significant decrease in blood levels of CXCL8 and IL-6 following 6 months of ETI treatment, as well as no overall changes in the functionality of blood neutrophils and monocytes collected from these patients (343).

Numerous studies have also evaluated the effects of CFTR modulator therapy on bacterial colonization in pwCF, particularly with *P. aeruginosa*. Results from the GOAL study indicated that 1 year of ivacaftor treatment significantly reduced the odds of *P. aeruginosa* culture positivity (by 35%), as well as the odds of *P. aeruginosa* being mucoid (by 23%), indicating a reduction in chronic infection burden (344). These changes may correspond with improved mucociliary clearance also observed in this study population, indicating some repair of this essential lung defense mechanism (117). However, another study by Harris et al. looking at subpopulations within the GOAL study, contrastingly observed minimal changes in *P. aeruginosa* colonization or bacterial load 6 months following ivacaftor therapy (345). They also reported overall minimal shifts in microbial communities, although, importantly, younger patients and patients without *P. aeruginosa* infection experienced greater shifts, suggesting that ivacaftor-induced changes in lung function may have greater ramifications for microbial colonization in patients with less-severe lung disease (345). A study in a separate cohort by Hisert et al. reported ivacaftor significantly reduced sputum concentrations of *P. aeruginosa* during the first year of therapy; however, patients still failed to eradicate their chronic *P. aeruginosa* infections and bacterial density rebounded after the first year, suggesting that ivacaftor therapy may not be sufficient to clear chronic *P. aeruginosa*, especially in patients with more severe lung disease (346). Recent studies investigating ETI therapy have shown more promising results. A study investigating 124 subjects receiving ETI therapy for at least 1 year reported that *P. aeruginosa* colonization decreased from approximately 54% of patients to 30% of patients (347), which has been corroborated by other studies (341). In a German CF cohort, it was recently shown that 21 months of ETI therapy overall decreased *P. aeruginosa* detection in throat swabs and sputum from approximately 40% to 23%, although it was less effective in older patients and those with lower baseline lung function (348), as previously reported for ivacaftor. However, the PROMISE study by Nichols et al. reported that while ETI therapy significantly decreased the sputum bacterial density of pathogens such as *P. aeruginosa*, *S. aureus*, and other species, those pathogens were still detectable by PCR despite sputum becoming culture-negative, indicating that infections still persisted at lower levels (349). This suggests that culture-based methods may not be sensitive enough to detect persisting pathogens. However, ETI treatment did increase overall bacterial diversity in sputum, which has also been reported by others (349, 350). ETI therapy has been shown to restructure the airway microbiome and metabolome, changing the metabolic niche in airway mucus to disfavor CF pathogens and decreasing the ratio of classical CF pathogens to anaerobes (350). Initiation of ETI therapy has also been shown to lead to a conversion in CF airway microbiomes from highly divergent and individualized before therapy toward a more similar and even community composition one year following therapy, suggesting a shift toward a healthier airway microbiome composition (351). Overall, these results suggest that HEMTs, particularly ETI therapy, have the potential to alter chronic *P. aeruginosa* infection in pwCF, decreasing but not entirely clearing *P. aeruginosa* from the lungs because of changes to the airway niche via improvements in CFTR function.

Overall, HEMTs, particularly with the approval of ETI therapy, are changing the landscape of CF disease and chronic *P. aeruginosa* infection and may potentially have broader impacts in non-CF chronic lung disease in future. Currently, trials are ongoing to confirm the safety of modulators in the infant, and even prenatal, populations of pwCF –those most likely to receive the greatest benefit from therapy in the form of attenuated development of structural lung disease and subsequent infections. However, there still exists a significant population of adult pwCF with established disease, including chronic inflammation and chronic infection, as well as a subset of patients (around 10%) who are ineligible for existing CFTR modulators. CFTR modulators have had variable success, thus far, at eradicating or lessening the burden of chronic *P. aeruginosa* infection in pwCF, with less success in those with more established lung disease (which often occurs because of *P. aeruginosa* infection itself). These populations would still benefit from the development of novel immunomodulatory therapies, and a deeper understanding of the host immune mechanisms rendering them susceptible to chronic infection with opportunistic pathogens like *P. aeruginosa*.

## 5 Discussion

The opportunistic pathogen *P. aeruginosa* has demonstrated a remarkable ability to colonize the lungs of individuals with chronic lung disease, particularly CF, as well as COPD and NCFB. Chronic infection leads to a robust yet ineffective inflammatory response that causes extensive and often irreversible lung damage leading to eventual lung failure. Chronic *P. aeruginosa* infection, for this reason, is strongly associated with worsened outcomes in individuals with chronic lung diseases. The inefficacy of the immune response to chronic *P. aeruginosa* is still not fully understood, although several key players have been identified. Neutrophils are recruited and sustained in high numbers during chronic infection via cytokine signaling which is skewed toward a particular proinflammatory milieu indicative of Th2 and Th17 immunity. Phagocytic and cytotoxic killing of *P. aeruginosa* is thwarted by various bacterial adaptations such as biofilm formation, LPS antigen modifications, loss of virulence factors such as flagella, and formation of persister cells, but also by impaired immune function in cells such as macrophages and neutrophils which may be secondary to chronic inflammatory stimuli and, in the case of CF, CFTR-intrinsic defects. B cells produce high levels of anti-*P. aeruginosa* IgG and IgA that also fail to provide appropriate protection. However, many questions remain. What are the roles of numerous other cell types in chronic *P. aeruginosa* infection? What key differences between acute and chronic infection render a response protective in one context but pathological in the other? Will differences between chronic *P. aeruginosa* infection in different chronic lung diseases require different treatment approaches?

With greater understanding of how the immune response to chronic *P. aeruginosa* is dysregulated, and how dysregulated host immunity may contribute to susceptibility to chronic *P. aeruginosa* infection, comes a greater ability to develop therapies able to modulate immunity against *P. aeruginosa* –either by dampening harmful inflammatory mediators such as neutrophil effectors, or by finding ways to boost immunity against *P. aeruginosa*, for example, through vaccine development. Many novel approaches to treat chronic *P. aeruginosa* and its associated inflammation have been developed, but comparatively few have found success, which may be a consequence of our still-incomplete knowledge. Even in the era of CFTR modulators, which can partially correct CFTR dysfunction and restore crucial aspects of lung function in pwCF, *P. aeruginosa* remains a threat. Current CFTR modulators appear unable to fully clear *P. aeruginosa* from patients already colonized, though they may reduce bacterial burden by improving mucociliary clearance and altering the lung microenvironment. However, CFTR modulator therapies are also generating new insights which will continue to advance our understanding of chronic *P. aeruginosa* respiratory infections, both in CF and other chronic lung diseases. As the landscape of chronic *P. aeruginosa* infection changes, with pwCF living longer lives and the prevalence of other chronic lung disease such as COPD and NCFB increasing, it is becoming both more conceivable and more pivotal to untangle the complex roles of host immunity in chronic *P. aeruginosa* infection.

## Author contributions

RN: Conceptualization, Writing – original draft, Writing – review & editing. CT: Writing – review & editing. BJ: Writing – review & editing. AL: Writing – review & editing. ZC: Conceptualization, Writing – original draft, Writing – review & editing.
